# Adeno-associated virus-mediated gene delivery promotes S-phase entry-independent precise targeted integration in cardiomyocytes

**DOI:** 10.1038/s41598-020-72216-y

**Published:** 2020-09-18

**Authors:** Yasuaki Kohama, Shuichiro Higo, Yuki Masumura, Mikio Shiba, Takumi Kondo, Takamaru Ishizu, Tomoaki Higo, Satoki Nakamura, Satoshi Kameda, Tomoka Tabata, Hiroyuki Inoue, Daisuke Motooka, Daisuke Okuzaki, Seiji Takashima, Shigeru Miyagawa, Yoshiki Sawa, Shungo Hikoso, Yasushi Sakata

**Affiliations:** 1 Department of Cardiovascular Medicine, Osaka University Graduate School of Medicine, 2-2 Yamadaoka, Suita, Osaka 565-0871 Japan; 2 Department of Medical Therapeutics for Heart Failure, Osaka University Graduate School of Medicine, Suita, Osaka 565-0871 Japan; 3 Higashiosaka City Medical Center, Higashiosaka, Osaka 578-8588 Japan; 4 Department of Medical Biochemistry, Osaka University Graduate School of Medicine, Suita, Osaka 565-0871 Japan; 5 Genome Information Research Center, Research Institute for Microbial Diseases, Osaka University, Suita, 565-0871 Japan; 6 Department of Cardiovascular Surgery, Osaka University Graduate School of Medicine, Suita, Osaka 565-0871 Japan

**Keywords:** Cardiovascular biology, DNA recombination, Genetic engineering

## Abstract

Post-mitotic cardiomyocytes have been considered to be non-permissive to precise targeted integration including homology-directed repair (HDR) after CRISPR/Cas9 genome editing. Here, we demonstrate that direct delivery of large amounts of transgene encoding guide RNA (gRNA) and repair template DNA via intra-ventricular injection of adeno-associated virus (AAV) promotes precise targeted genome replacement in adult murine cardiomyocytes expressing Cas9. Neither systemic injection of AAV nor direct injection of adenovirus promotes targeted integration, suggesting that high copy numbers of single-stranded transgenes are required in cardiomyocytes. Notably, AAV-mediated targeted integration in cardiomyocytes both in vitro and in vivo depends on the Fanconi anemia pathway, a key component of the single-strand template repair mechanism. In human cardiomyocytes differentiated from induced pluripotent stem cells, AAV-mediated targeted integration fluorescently labeled Mlc2v protein after differentiation, independently of DNA synthesis, and enabled real-time detection of sarcomere contraction in monolayered beating cardiomyocytes. Our findings provide a wide range of applications for targeted genome replacement in non-dividing cardiomyocytes.

## Introduction

Genomic cleavage after DNA double-strand breaks (DSB) induced by CRISPR-mediated genome editing is repaired through the non-homologous end-joining (NHEJ) or homology-directed repair (HDR) pathways^1–3^. In contrast to NHEJ, which is an error-prone process that forms an insertion or deletion at the DSB site, HDR enables accurate genome repair using single- or double-stranded DNA templates. Because HDR primarily occurs during the S/G2 phase, the HDR-based repair process after genome editing is restricted to cells that are actively dividing^4–7^, thereby limiting its application to hearts consisting of non-dividing cardiomyocytes that cease to proliferate after birth^8^. Previously we demonstrated that Adeno-associated virus (AAV)-mediated gene delivery enabled targeted genome replacement via HDR, independently of DNA synthesis, in non-dividing cultured cardiomyocytes^9^ isolated from Cas9 knock-in mice^10^. Transduction of AAV6 encoding gRNA and a repair template efficiently introduced the tdTomato sequence into the 3′ end of the *Myl2* gene encoding Mlc2v, a ventricular isoform of myosin regulatory light chain, and yielded the sarcomeric Mlc2v-tdTomato fusion protein. Although Mlc2v plays a pivotal role in cardiogenesis and sarcomeric function^11,12^, the introduction of tdTomato fluorescent protein into the C-terminus of endogenous Mlc2v protein does not significantly affect sarcomere structure^9,13^, suggesting that *Myl2* is a useful and appropriate endogenous target gene to detect HDR or visualize sarcomere contraction in cardiomyocytes. Although our previous findings demonstrated that AAV-mediated gene delivery promoted HDR in cardiomyocytes that had not entered S-phase, these observations were limited to cultured conditions using murine cardiomyocytes in the neonatal stage. Although CRISPR/Cas9-mediated targeted gene disruption via NHEJ has thus far been effective in murine adult heart tissues^14^, HDR-mediated replacement of the targeted genome in cardiomyocytes of the adult heart has not been reported. Furthermore, the mechanism underlying targeted integration in non-dividing cardiomyocytes remains to be elucidated.

Here we demonstrated that AAV-mediated gene delivery promoted precise targeted integration in murine adult heart tissues and human cardiomyocytes after differentiation from induced pluripotent stem cells (hiPSCs) independently of DNA synthesis, and enabled direct visualization of sarcomeres. In contrast to AAV, adenovirus (AdV) promoted targeted integration only in a limited population of cultured neonatal cardiomyocytes, all of which had entered S-phase, suggesting a distinct underlying molecular mechanism. We clarified that targeted integration promoted in non-dividing cardiomyocytes was mediated by the recently identified Fanconi anemia pathway involved in single-strand template repair (SST-R)^15^. Our findings uncover the essential role of AAV-mediated gene delivery in promoting precise integration in cardiomyocytes and suggest a wide range of applications for targeted genome replacement.

## Results

### AAV-mediated gene delivery promotes precise targeted integration in adult cardiomyocytes

**Figure 1:**
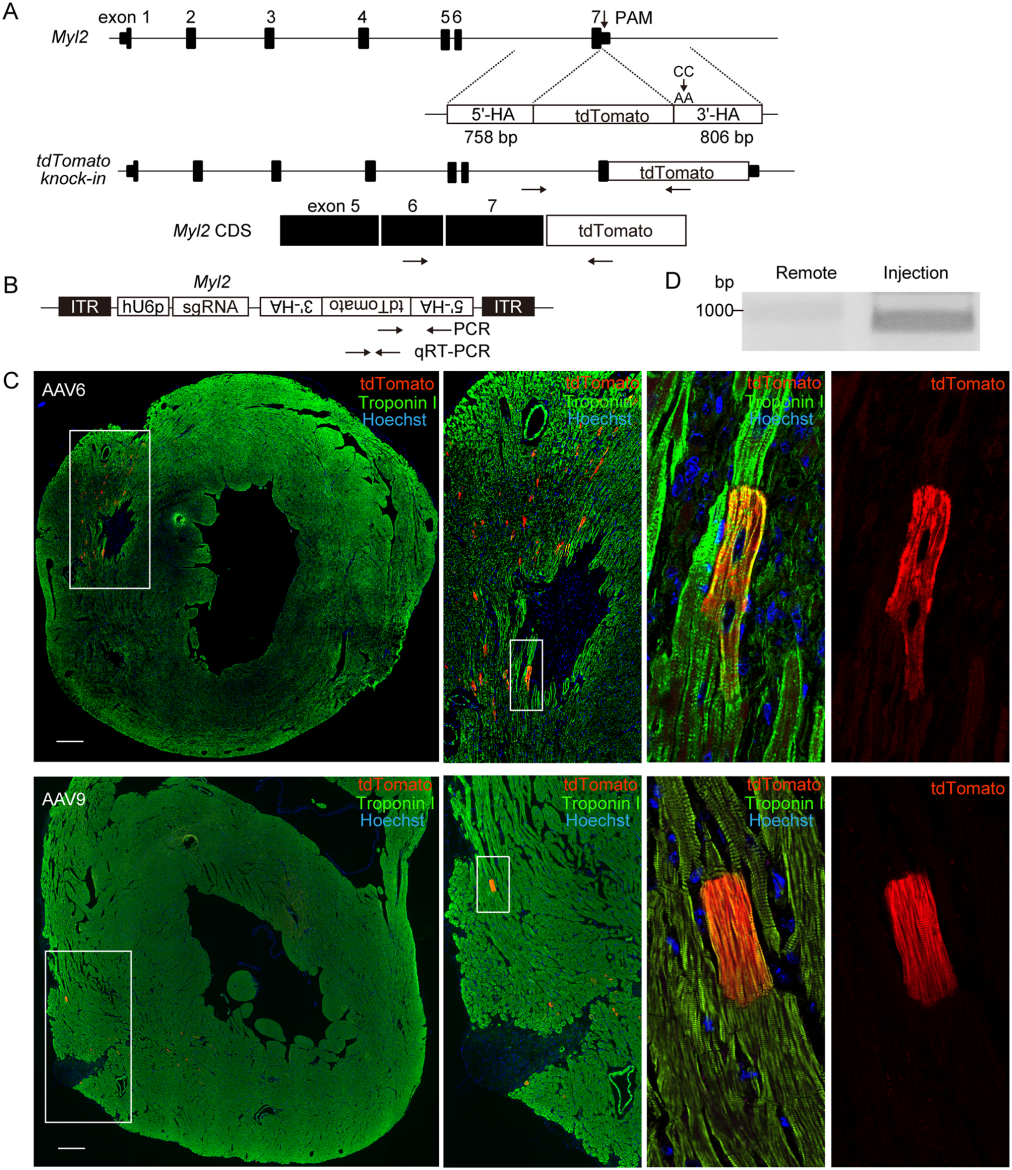
Direct injection of AAV-induced precise targeted integration in adult murine heart tissues. (**A**) Genomic sequences of mouse *Myl2* and repair template (upper) are shown, along with the expected genomic sequence after targeted integration (middle) and the 3′-terminal coding sequence (CDS) of *Myl2* (lower). The repair template consisting of the coding sequence of the tdTomato fluorescent reporter protein between the 758-bp 5′-terminal and the 806-bp 3′-terminal homology arms (5′-HA and 3′-HA) corresponding to the genomic sequence of the 3′-terminus of the *Myl2*. PAM mutation (CC to AA) was introduced into 3′-HA, and the stop codon sequence was removed. Arrows indicate the locations of PCR primers to detect targeted integration in genomic DNA (middle) or in cDNA (lower). The forward primer to amplify genomic sequences was designed outside of 5´-HA. The primer pairs to amplify CDS were designed to span the intronic sequence. (B) The DNA sequence containing hU6 promoter (hU6p)–driven gRNA and a tdTomato repair template in the opposite direction was subcloned into AAV6 or AAV9. Arrows indicate the locations of primers to amplify the transgene by PCR and quantitative real-time PCR (qRT-PCR). (**C**) AAV6 (2.5 × 10^9^ viral genome/10 μl) or AAV9 (9.2 × 10^10^ viral genome/10 μl) was directly injected into the free left ventricular wall. Five days after injection, heart tissues were excised and stained with anti-troponin I antibody (green). Scale bar: 300 μm. (**D**) Intron-spanning PCR was performed using cDNA samples obtained from AAV6-injected myocardium. cDNA obtained from remote sections that did not undergo viral injection were used as controls. Original full image of the gel is presented in Supplementary Fig. 6A.

To induce HDR in cardiomyocytes, we previously generated AAV6 encoding gRNA targeting the 3′ end of the mouse *Myl2* gene and repair template DNA encoding tdTomato fluorescent protein flanked by homology arms (Fig. 1A). The generated vector efficiently replaced the target genome and yielded C-terminally tagged Mlc2v-tdTomato fusion protein in cultured neonatal cardiomyocytes^9^ isolated from Cas9 knock-in mice in which 3xFLAG-fused Cas9 and a P2A self-cleavable peptide followed by EGFP protein were knocked in at the endogenous *Rosa26* locus^10^. In our previous experiments, transduction of AAV6 into cardiomyocytes isolated from murine adult heart tissue by the Langendorff perfusion method failed to induce HDR^9^, probably because of the low transduction efficiency or the low viability of isolated adult cardiomyocytes after viral transduction. Because AAV9, like AAV6, achieves high transduction efficiency in rodent hearts^16^, we generated AAV9 encoding the same gene components (Fig. 1B), and confirmed Mlc2v-tdTomato–positive cardiomyocytes after viral transduction in in vitro conditions (Supplementary Fig. 1A). To promote HDR in in vivo heart tissue, AAV6 or AAV9 was systemically injected via the orbital venous sinus into 8-week-old Cas9 knock-in mice. Although the transgenes were detected in myocardium 7 days after injection (Supplementary Fig. 1B), tdTomato-positive cardiomyocytes were not observed in either AAV6- or AAV9-injected mouse hearts (Supplementary Fig. 1C). To increase the copy number of transgenes, we directly injected AAV6 (2.5 × 10^9^ viral genomes) or AAV9 (9.2 × 10^10^ viral genomes) encoding gRNA and repair template DNA into the left ventricular myocardium of 8-week-old Cas9 knock-in mice. Intriguingly, 5 days after injection, tdTomato-positive cardiomyocytes were observed scattered around the injection site in the left ventricular myocardium of both AAV6- and AAV9-injected mouse hearts (Fig. 1C). Troponin I staining showed that tdTomato fluorescent signals were merged with the sarcomere structure. Intron-spanning PCR using cDNA samples obtained from injected myocardium and Sanger sequence analysis confirmed that the tdTomato sequence was precisely introduced into the 3′-terminus of the *Myl2* gene (Fig. 1D and Supplementary Fig. 1D). We evaluated whether AAV mediates precise targeted integration without Cas9 in our experimental conditions, because AAV mediates homologous recombination in the absence of exogenous nucleases^17^. We directly injected AAV6 encoding gRNA and repair template DNA into the left ventricular myocardium of 8-week-old WT mice. Five days after injection, tdTomato-positive cardiomyocytes were not observed in the dissected tissues (Supplementary Fig. 1E), suggesting that precise integration of tdTomato into the 3′-terminus of *Myl2* in adult heart tissues required DSB caused by Cas9. To determine whether genomic replacement actually occurred in cardiomyocytes without S-phase entry in ventricular tissues injected with AAV6, 5-ethynyl-2′-deoxyuridine (EdU)^18^ was injected to mice 2 h before AAV6 transduction, then injected for 5 consecutive days after transduction (Supplementary Fig. 1F). Five days after AAV6 transduction, EdU-positive non-cardiomyocytes were observed around the injection site (Supplementary Fig. 1G), representing the reactive proliferation of interstitial cells after injury and the successful incorporation of the injected EdU. tdTomato-positive cardiomyocytes were observed around the injection site 5 days after injection, and none of these cells were positive for EdU (Supplementary Fig. 1G). These data suggest that direct injection of AAV induced precise targeted integration in cardiomyocytes in adult murine heart tissues independently of S-phase entry.

### A high transgene copy number is required to promote targeted integration

**Figure 2:**
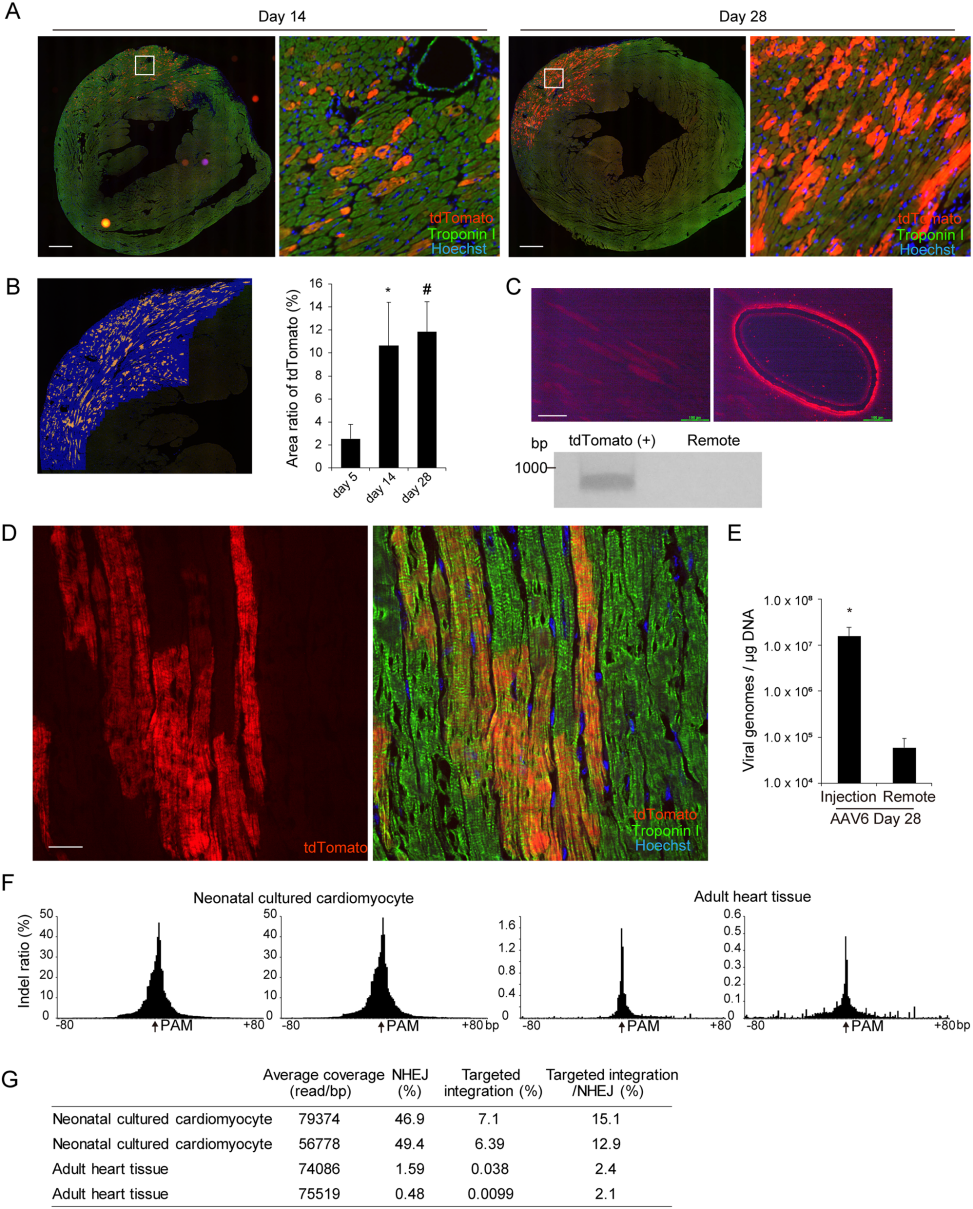
A high transgene copy number is required to promote targeted integration in adult cardiomyocytes. (**A**) AAV6 was directly injected into the free left ventricular wall. At 14 and 28 days after injection, heart tissues were excised and stained with anti-troponin I antibody (green). Scale bar: 300 μm. (**B**) The proportion of the injection area in the myocardium that was tdTomato positive, as estimated by the distribution of tdTomato fluorescent signals, were calculated using BZ-X analyzer (Keyence) (n = 3, means ± SD). *: p < 0.05, #: p < 0.01 vs day 5. (**C**) The area of a frozen tissue section positive for tdTomato at day 28 after injection was excised using laser micro dissection. Cross-sections before (left) and after (right) dissection are shown. Scale bar: 100 μm. A remote area was excised as control. RNA was extracted from both sections and intron-spanning PCR was performed using the cDNA samples. Original full image of the gel is presented in Supplementary Fig. 6B. (**D**) Tissue sections obtained at day 28 after AAV injection were immunostained and observed by confocal microscopy. Scale bar: 20 μm. (**E**) Genomic DNA was extracted from the dissected tissue sections and quantitative real-time PCR was performed. Forward and reverse primers were designed to amplify the ITR sequence of AAV. The numbers of transgenes were calculated as ITR copy numbers normalized by the amount of genomic DNA used as the PCR template. (**F**) Neonatal cultured cardiomyocytes isolated from Cas9 knock-in mice were transduced with AAV6 encoding gRNA and repair template DNA. Five days after transduction, genomic DNA were extracted. Left ventricular free wall of 8-week old Cas9 knock-in mice were injected with AAV6. Five days after injection, genomic DNA were extracted. Both experiments were performed in duplicate. The PCR-amplified genomic DNA were analyzed by deep sequencing. Indel ratio normalized by the average coverage around the PAM sequence are shown. (**G**) Average coverage (read/bp), ratio of NHEJ, targeted integration and proportion of targeted integration/NHEJ were analyzed in neonatal cultured cardiomyocytes and adult heart tissues.

We obtained myocardial tissues 14 and 28 days after direct injection of AAV6. Of note, cardiomyocytes positive for tdTomato fluorescent signals localized at sarcomeres were significantly increased around the injection site at day 14, and these tdTomato-positive cardiomyocytes remained intact until day 28 (Fig. 2A and B). To confirm that Myl2-tdTomato fusion mRNA was transcribed in local tdTomato-positive sections at day 28, total RNA was extracted from tdTomato-positive and -negative micro tissue sections obtained by laser microdissection. Intron-spanning PCR and Sanger sequencing analysis using cDNA samples confirmed precise genome replacement (Fig. 2C and Supplementary Fig. 2A). Confocal microscopy analysis demonstrated that Mlc2v-tdTomato protein precisely visualized sarcomeric A-bands tandemly aligned with I-bands detected by troponin I staining in the heart tissues 28 days after direct injection (Fig. 2D and Supplementary Fig. 2B). These data suggest that Mlc2v-tdTomato fusion protein was gradually replaced with endogenous Mlc2v protein after viral transduction, and did not significantly affect sarcomere structure in the heart in vivo. To determine the copy number of the local transgenes delivered by direct injection of AAV6, genomic DNA was obtained from dissected cardiac sections at day 28, including from tdTomato-positive cardiomyocytes at injected sites and tdTomato-negative cardiomyocytes at remote sites (Supplementary Fig. 2C). Notably, although hemodynamic delivery after direct injection caused the transgenes in remote sites to be detected by PCR analysis at day 28 (Supplementary Fig. 2D), tdTomato-positive cardiomyocytes could not be detected there. Quantitative real-time PCR analysis clarified that the copy number of transgenes in the injected sections containing tdTomato-positive cardiomyocytes were more than 100-fold higher than those in the remote sections (Fig. 2E). These data suggest that Mlc2v-tdTomato fusion protein in cardiomyocytes was robustly expressed 28 days after transduction, and that a high copy number of the transgenes are required to promote targeted integration in adult murine cardiomyocytes in vivo.

Although immunostaining experiments clarified the precise genome replacement in adult heart tissues, the other outcomes including NHEJ-mediated deletion or insertion were also expected. To evaluate this issue, genomic DNA were extracted both from neonatal cultured cardiomyocytes and adult heart tissues of Cas9 knock-in mice treated with AAV6 encoding gRNA and repair template. The targeted DNA sequences were amplified with the primes outside the both 5′- and 3′- homology arms and analyzed by the targeted deep sequencing in the duplicate samples. Both in neonatal cultured cardiomyocytes and adult heart tissues, indel mutations caused by NHEJ were detected around the PAM sequence targeted by the gRNA (Fig. 2F). Deep sequence analysis with extremely high coverage demonstrated that NHEJ and targeted integration (knock-in of tdTomato) was detected in cultured neonatal cardiomyocytes at a rate of 46.9%, 7.1% and 49.4%, 6.39%, respectively. In adult heart tissues, NHEJ and targeted integration were detected at a rate of 1.59%, 0.038% and 0.48%, 0.0099%, respectively (Fig. 2G). Notably, the efficiency of targeted integration compared to NHEJ estimated by deep sequencing were significantly lower in adult heart tissues than in neonatal cultured cardiomyocytes (15.1%, 12.9% in neonatal cultured cardiomyocytes, and 2.4%, 2.1% in adult heart tissues, Fig. 2G).

### AdV does not promote S-phase entry-independent targeted integration

**Figure 3:**
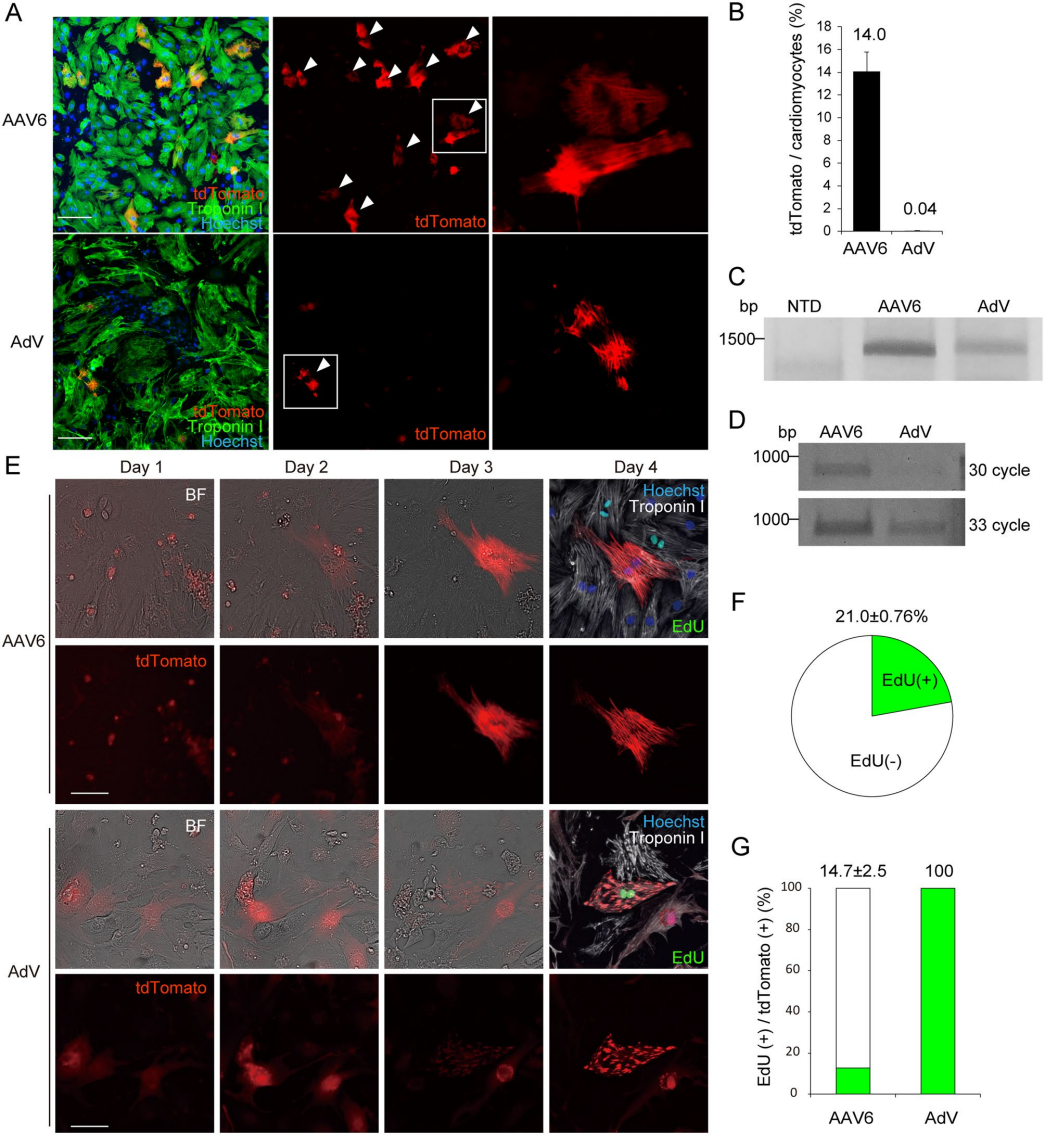
Imaging-based evaluation of targeted integration in cardiomyocytes transduced with AAV or AdV. (**A**) Neonatal cardiomyocytes isolated from Cas9 knock-in mice were seeded in 96-well plates and transduced with AAV6 (8.3 × 10^4^ viral genomes/cell) or AdV (1.4 × 10^10^ PFU/ml, 1 μl/well). Five days after transduction, cells were fixed and stained with anti-troponin I antibody (green). Cardiomyocytes in which tdTomato signal co-localized with sarcomeres are indicated with arrowheads. Scale bar: 100 μm. (**B**) The proportion of tdTomato-positive cardiomyocytes was calculated using high-content image analysis (AAV6, n = 6, AdV, n = 8, means ± SD). (**C**) Cardiomyocytes were treated as in (A). Four days after transduction, genomic DNA was extracted and the targeted sequence was amplified by PCR using the primers indicated in Fig. 1A. Original full image of the gel is presented in Supplementary Fig. 6C. (**D**) Cardiomyocytes were treated as in (A). Four days after transduction, total RNA was extracted and converted to cDNA. The targeted coding sequence was amplified by PCR using the primers indicated in Fig. 1A. Original full image of the gel is presented in Supplementary Fig. 6D. (**E**) Cardiomyocytes seeded in 96-well plates were transduced with AAV6 or AdV at 6 h after the addition of 5 μM EdU, followed by continuous labeling for 4 days. After viral transduction, bright-field (BF) and fluorescence images were sequentially obtained using high-content image analysis targeting the same fields determined by the coordinate axes. At day 4, cardiomyocytes were fixed and immunostained with anti-troponin I antibody (white). Representative sequential images of cardiomyocytes positive for tdTomato are shown. Scale bar: 50 μm. (**F**) Cardiomyocytes were treated with 5 μM EdU continuously for 4 days. Then cells were fixed and immunostained. The proportions of EdU-positive cardiomyocytes were determined (n = 3, means ± SD). (**G**) Cardiomyocytes were treated as in (C). The proportion of tdTomato-positive cardiomyocytes that were also positive for EdU was calculated using high-content image analysis (n = 3, means ± SD).

AAV is a nonenveloped virus with single-stranded DNA (ssDNA), and imported ssDNA is converted to double-stranded DNA and transcribed^19^. To determine whether gene delivery as ssDNA is required to promote targeted integration in cardiomyocytes, we used adenovirus (AdV) that deliver transgenes as double-stranded DNA and achieve high transduction efficiency and robust expression of the transgene in cardiomyocytes^20^. We first validated the ability of AdV to promote targeted integration in cultured cardiac fibroblasts isolated from neonatal Cas9 knock-in mice. AdV encoding gRNA and repair template DNA successfully introduced the tdTomato sequence into the 3′ end of the mouse *Actb* gene (Supplementary Fig. 3A–E). Next, to investigate whether AdV-mediated gene delivery also promote targeted integration in cardiomyocytes, the gRNA and repair template sequence targeting *Myl2* located between the inverted terminal repeat (ITR) sequence (Fig. 1B) was entirely subcloned into the cloning vector to produce AdV. To determine whether AdV-mediated gene delivery also promote targeted integration in adult murine heart tissue, we directly injected AdV into the left ventricular myocardium of 8-week-old Cas9 knock-in mice. Five days after AdV injection, tdTomato fluorescent signals were undetectable (Supplementary Fig. 3F) despite the comparable transduction efficiencies of AdV and AAV6 transgenes in heart tissues (Supplementary Fig. 3G). To further evaluate the characteristics of both viral vectors, we transduced both AAV6 and AdV encoding the same sequence into cultured neonatal cardiomyocytes isolated from Cas9 knock-in mice and performed sequential observations using high-content image analysis. Transduction at the adjusted viral titer led to comparable transduction efficiencies between AAV and AdV as determined by quantitative real-time PCR (Supplementary Fig. 3H). After transduction with AAV6, cardiomyocytes positive for tdTomato fluorescent signals clearly localized in the sarcomere were observed at a rate of 14% at day 5 (Fig. 3A and B), consistent with previous findings^9^. After transduction by AdV encoding the same DNA sequence, only a limited number of cardiomyocytes positive for tdTomato fluorescent signals localized in the sarcomere were detected, (Fig. 3A, arrowhead), at a rate of 0.04% (Fig. 3B). PCR analysis using genomic DNA and cDNA followed by Sanger sequencing validated the precise integration at the *Myl2* gene mediated by both viral vectors (Fig. 3C, D and Supplementary Fig. 3I), although the efficiency of targeted integration was significantly lower in cardiomyocytes transduced with AdV than with AAV, consistent with the immunostaining results. To clarify the characteristics of the limited population of tdTomato-positive cardiomyocytes transduced with AdV, we investigated the association between targeted integration and DNA synthesis in cardiomyocytes. EdU was added to cardiomyocytes 6 h before transduction of AAV or AdV, then cardiomyocytes were continuously labeled for 4 days. Cardiomyocytes were sequentially observed, and the images of immunostained samples fixed on day 4 were quantitatively analyzed using high-content image analysis (Fig. 3E). After 4-day culture, 21% of cardiomyocytes were positive for EdU, suggesting that these cells had entered S-phase at least once (Fig. 3F). AAV-mediated gene delivery uniformly promoted targeted integration both in cardiomyocytes that had and had not passed S-phase (Fig. 3E and G), as previously reported^9^. In contrast, in cardiomyocytes transduced with AdV, the small number of cardiomyocytes positive for tdTomato fluorescent signals that co-localized with sarcomeres were all double positive for EdU (Fig. 3E and G). These data suggest that targeted integration promoted by AdV transduction was restricted in cardiomyocytes that had entered S-phase, whereas AAV-mediated gene delivery promoted targeted integration independently of S-phase entry.

### Inhibition of the Fanconi anemia pathway suppresses targeted integration in cardiomyocytes

**Figure 4:**
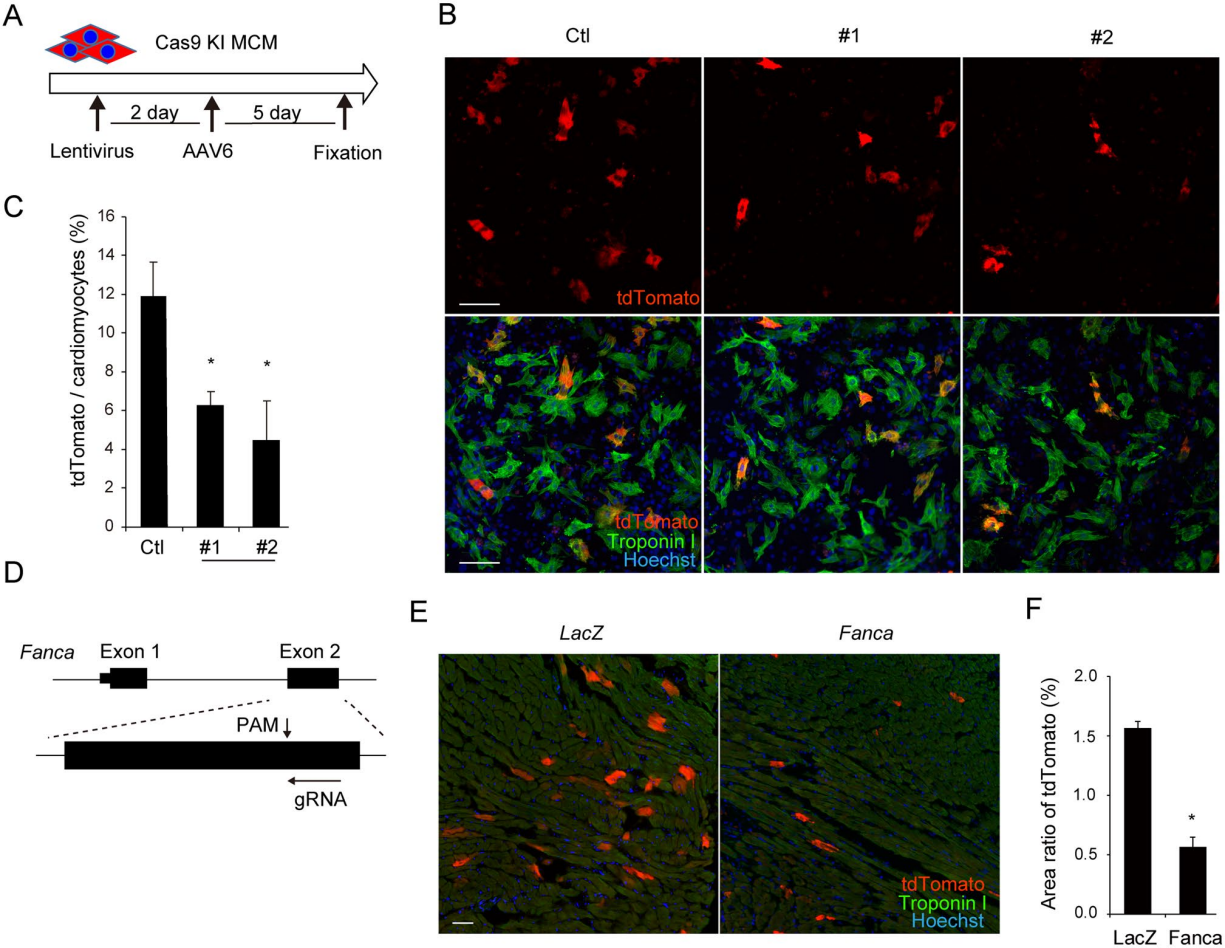
Inhibition of the Fanconi anemia pathway suppresses AAV-mediated targeted integration in cardiomyocytes. (**A**) Experimental procedure of *Fanca* inhibition. Neonatal cardiomyocytes isolated from Cas9 knock-in mice were seeded in 96-well plates and transduced with lentivirus encoding shRNA against *Fanca*. Two days after shRNA transduction, cardiomyocytes were transduced with AAV6. Five days after AAV6 transduction, cells were fixed and immunostained. (**B**) Cardiomyocytes were treated as in (A). Cells were stained with anti-troponin I antibody (green). Scale bar: 100 μm. (**C**) The proportion of tdTomato-positive cardiomyocytes was calculated using high-content image analysis (n = 3, means ± SD). *: p < 0.05 vs control. (**D**) gRNA was designed targeting exon 2 of the mouse *Fanca* gene. (**E**) AAV6 at 2.5 × 10^9^ viral genome encoding gRNA targeting *Fanca* or *Lacz* was co-injected with AAV6 at 2.5 × 10^9^ viral genome encoding gRNA and repair template targeting *Myl2* into myocardium of 8-week-old Cas9 knock-in mice. Seven days after injection, heart tissues were excised. The sections of heart tissue were fixed and stained with anti-troponin I antibody (green). Scale bar: 50 μm. (**F**) The proportion of the injection area in the myocardium that was tdTomato positive, as estimated by the distribution of tdTomato fluorescent signals, were calculated using BZ-X analyzer (Keyence) (n = 3, means ± SD). *: p < 0.01.

HDR includes three pathways: homologous recombination (HR), microhomology-mediated end joining (MMEJ), and single-strand template repair (SST-R)^21^. HR uses homologous sequences of sister chromatids or exogenously provided DNA sequences as repair templates^4^. MMEJ repairs DSB via short homology sequences containing a few complementary nucleotides^22^. SST-R repairs DSB using single-strand oligo DNA, which has high efficiency in human cells^23^. In contrast to the former two pathways, the underlying mechanism of SST-R is poorly understood. A recent study demonstrated that SST-R after Cas9-mediated DSB required the Fanconi anemia (FA) pathway, which is normally involved in the mechanism of repairing interstrand cross-links^15^. To investigate whether AAV-mediated targeted integration in cardiomyocytes requires the FA pathway, *Fanca*, a key component of the FA pathway^15,24^, was knocked down using shRNA (Fig. 4A). Lentivirus-mediated delivery of shRNAs significantly decreased the mRNA expression levels of *Fanca* in cultured neonatal cardiomyocytes (Supplementary Fig. 4A). As shown in Fig. 4B and C, knockdown of *Fanca* using two different shRNAs significantly reduced the number of tdTomato-positive cardiomyocytes after AAV transduction. To further determine the in vivo relevance of the FA pathway, we generated AAV6 encoding gRNA that targeted exon 2 of the *Fanca* gene (Fig. 4D). Transduction of AAV6 into cultured cardiomyocytes isolated from neonatal Cas9 knock-in mice induced NHEJ at the targeted locus (Supplementary Fig. 4B) and significantly decreased *Fanca* mRNA expression levels (Supplementary Fig. 4C). The myocardium of 8-week-old Cas9 knock-in mice were co-injected with AAV6 encoding *Fanca*-targeting gRNA and AAV6 encoding gRNA with a repair template targeting *Myl2*. At day 7 after direct injection, the number of tdTomato-positive cardiomyocytes was significantly decreased in myocardium co-injected with AAV6 targeting *Fanca* compared to AAV6 targeting *LacZ* (Fig. 4E and F). *BRCA2*, also known as *FANCD1*, is involved in FA pathway via regulation of homologous recombination^24^, and is required for AAV-mediated homologous recombination in B lymphoblastoid cell lines^17^. As shown in Supplementary Fig. 4D and E, knock down of *Brca2* significantly reduced the number of tdTomato-positive cardiomyocytes after AAV transduction, suggesting that *Brca2* as well as *Fanca* play pivotal roles in the AAV-mediated targeted integration in cardiomyocytes. These data suggest that targeted integration promoted by AAV transduction at least partly depends on an SST-R mechanism mediated by the Fanconi anemia pathway.

### AAV-mediated targeted integration is effective in differentiated hiPSC-CMs

**Figure 5:**
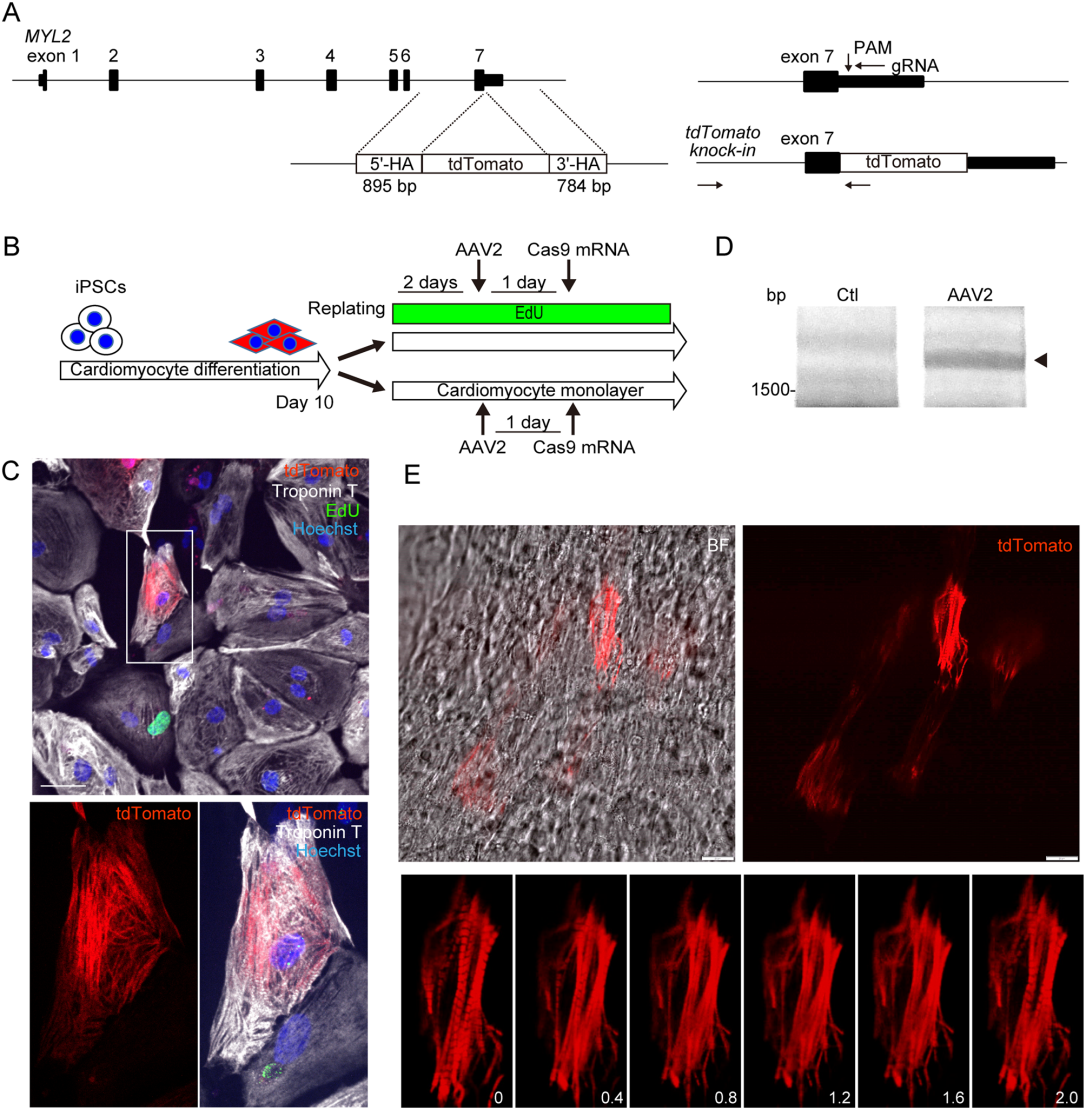
AAV2 transduction followed by transient expression of Cas9 promotes targeted genome replacement in human monolayered cardiomyocytes differentiated from hiPSCs. (**A**) Genomic sequence of Human *MYL2*. gRNA was designed in an antisense direction targeting the PAM sequence located 19 bp downstream of the MYL2 stop codon. The repair template consists of the coding sequence of the tdTomato fluorescent reporter protein between the 895-bp 5′-terminal and the 784-bp 3′-terminal homology arms (5′-HA and 3′-HA), corresponding to the genomic sequence of the 3′-terminus of *MYL2*. Arrows indicate the locations of PCR primers to detect targeted integration after successful recombination. The forward primer to amplify the genomic sequence was designed outside of 5′-HA. (**B**) Experimental procedure of cardiomyocyte differentiation from hiPSCs and AAV2 transduction. hiPSCs were differentiated into cardiomyocytes using a monolayer protocol. Ten days after differentiation induction, hiPSC-CMs were replated into 96-well plates. EdU was added 2 days before AAV2 transduction. One day after transduction, cardiomyocytes were transfected with mRNA encoding Cas9, then continuously incubated with EdU. Five days after transduction, cells were fixed and immunostained. (**C**) A representative image of a tdTomato-positive, EdU (green)-negative, Troponin T (white)-positive hiPSC-CM is shown. Scale bar: 50 μm. (**D**) hiPSC-CMs were treated as in (**B**). Seven days after AAV2 transduction, genomic DNA was extracted and the targeted sequence was amplified by PCR using the primers indicated in (A). Original full image of the gel is presented in Supplementary Fig. 6E. (**E**) Contracting, monolayered, sheet-like hiPSC-CMs 14 days after differentiation were transduced with AAV2, then transfected with mRNA encoding Cas9. Scale bar: 20 μm. Lower panels show the serial consecutive fluorescent images obtained every 0.4 s.

Finally, we examined whether AAV-mediated targeted integration is effective in human cardiomyocytes, because a direct method to replace the targeted genome in differentiated, contracting human cardiomyocytes may provide a variety of analytical applications. To this end, we differentiated the hiPSCs generated from a cardiomyopathy patient into cardiomyocytes (hiPSC-CMs) using a 2D monolayer method as previously described^25^. This yielded approximately 84% troponin T–positive cardiomyocytes at day 10 after differentiation induction (Supplementary Fig. 5A). We designed gRNA targeting the 3′ end of the human *MYL2* gene and repair template DNA encoding tdTomato fluorescent protein flanked by the homology arms (Fig. 5A). gRNA was designed in an antisense direction targeting a PAM sequence located 19 bp downstream of the *MYL2* stop codon to avoid Cas9-mediated re-cleavage of exon 7. We generated AAV2 encoding a gRNA sequence and repair template, because AAV2 was previously shown to efficiently transduce hiPSC-CMs^26^, and was found to transduce hiPSC-CMs with high efficiency in our experimental conditions (Supplementary Fig. 5B). The differentiated cardiomyocytes were trypsinized and replated onto a 96-well plate at day 10 after differentiation. These cells were labeled with EdU, then transduced with AAV2 encoding gRNA and a repair template, followed by transfection with mRNA encoding Cas9 (Fig. 5B). Continuous labeling with EdU demonstrated that after replating, approximately 5% of the troponin T–positive hiPSC-CMs entered S-phase during 3-day culture (Supplementary Fig. 5C and 5D). Sequential observation using high-content image analysis detected MLC2v-tdTomato–positive hiPSC-CMs without S-phase entry (Fig. 5C). Precise targeted integration was confirmed by genomic PCR and Sanger sequencing analysis (Fig. 5D and Supplementary Fig. 5E). Based on these results, we directly transduced AAV2 into contracting, monolayered, sheet-like hiPSC-CMs at day 14 (Fig. 5B), then transfected mRNA encoding Cas9. Seven days after transfection, fluorescent-labeled sarcomeres were visible in monolayered hiPSC-CMs, enabling live tracing of the oriented sarcomeric A-bands in hiPSC-CMs (Fig. 5E and Supplementary movie). These data suggest that AAV2-mediated gene delivery combined with transient transfection of Cas9 promoted targeted integration in human monolayered hiPSC-CMs and allowed direct visualization of contracting sarcomeres after differentiation.

## Discussion

Precise genome replacement in non-dividing cardiomyocytes has been challenging because the repair process using HDR is restricted to dividing cells^4–7^. The recently developed homology-independent targeted integration (HITI) strategy allows for high-efficiency DNA knock-in in post-mitotic cells and enables effective knock-in of the fluorescent reporter in in vivo murine heart tissues at postnatal day 1^27^. However, HITI replaces the targeted genome via a NHEJ-based mechanism, and thereby potentially causes insertion or deletion mutations at the junction site after successful knock-in. Furthermore, techniques for precise genome replacement in adult murine heart tissues have not yet been developed. In this report we demonstrated for the first time that AAV-mediated gene delivery promotes precise targeted integration in cardiomyocytes in in vivo murine adult heart tissues independently of DNA synthesis. In spite of the comparable transduction efficiency in cardiomyocytes, AdV-mediated gene delivery does not promote targeted integration in cardiomyocytes without S-phase entry. These data suggest that AAV-mediated gene delivery is the critical determinant for promoting targeted integration in non-dividing cardiomyocytes, consistent with previous findings that demonstrated AAV-mediated HDR in post-mitotic neuronal cells^28^.

AAV contains a transgene flanked by inverted terminal repeats, and the transgene is introduced into infected cells as a ssDNA^18^. Among the repair pathways after DNA double-strand breaks, the SSTR pathway using ssODN is highly effective in human cell lines^29^ and human hematopoietic stem cells^30^ . A recent report using CRISPR inhibition screening clarified that SSTR after genome editing required the FA pathway, and knockdown of the genes involved in this pathway, including *FANCA*, significantly decreased the efficiency of SSTR in K562 cells^15^. In our experiments, knockdown of *Fanca* significantly decreased the efficiency of targeted integration both in cultured cardiomyocytes and in vivo in the heart. FANCA promotes DNA double-strand break repair via single-strand annealing or strand exchange, and mutant FANCA protein with genetic variants of FA patients demonstrates defective biochemical activity in single-strand annealing or strand exchange^31^. Because FA pathway is generally considered to be a repair mechanism targeting interstrand crosslinks^24^, genomic structure formed during the repairing process in cardiomyocytes using ssDNA introduced by AAV as a transgene may resemble the conformation targeted by FA pathway. In our experimental conditions, knockdown of *Brca2* decreased the efficiency of targeted integration in cultured cardiomyocytes. Although *Fanca* and *Brca2* are ubiquitously expressed in various tissues, the decreased efficiency of AAV-mediated targeted integration in cardiomyocytes via knockdown of these genes suggests that SSTR-mediated DNA repair plays a pivotal role in terminally differentiated cardiomyocytes.

Introduction of the tdTomato sequence into the 3′-terminus of the *Myl2* gene did not significantly injure cardiomyocytes in vitro 6 days after transduction of AAV6^9^, or heart tissues in vivo 28 days after direct injection of AAV6. These data suggest that Mlc2v-tdTomato fusion protein produced by the recombined genetic locus did not significantly affect sarcomere function, and the *Myl2* gene might be an appropriate target for real-time imaging of sarcomere structure even in the in vivo heart. A recent genome editing study using human hiPSCs demonstrated that fluorescent gene tagging of the genes involved in sarcomere function (*TTN*, *MYL7*, *TNNI1*, *ACTN2*, and *MYL2*) allowed live visualization of their proteins, which can potentially provide a useful research tool targeting cardiac morphogenesis^13^. Although these methods are useful for visualization and functional analysis of sarcomere proteins, the consecutive steps from genome editing to clonal selection of the edited hiPSCs is required. Furthermore, image-based validation of successful recombination must be confirmed after cardiomyocyte differentiation from hiPSCs, and is thereby time-consuming. Our method rapidly introduced the tdTomato sequence into the 3′-terminus of the *MYL2* gene in differentiated contracting cardiomyocytes, and therefore this technique can potentially be used in various disease-specific hiPSC-CMs.

Rapid advances in high-throughput sequencing technologies have confirmed the high incidence of pathological genetic mutations in hereditary cardiomyopathies that lead to advanced heart failure^32–35^. CRISPR/Cas9 genome editing technologies are now generally recognized as potential tools for directly correcting genetic mutations^6,36^. HDR is a promising mechanism to correct pathogenic gene mutations, and is thereby expected to be a therapeutic tool for intractable cardiomyopathies. However, at this stage, the therapeutic application of targeted genome replacement in heart tissues is associated with several challenging problems that have yet to be overcome. First, our experiments indicate that the introduction of a high copy number of transgenes is required to promote targeted integration in the murine heart in vivo. Gene replacement therapy using AAV1 encoding SERCA2a (sarcoplasmic/endoplasmic reticulum Ca^2+^-ATPase) into the human heart via intracoronary administration failed to improve the clinical course of patients with heart failure^37^. The major reason for the negative results of the clinical study is presumed to be the low amount of vector DNA detected in these tissues, suggesting that it may be difficult to establish an efficient delivery system to transfer the target gene into heart tissues. Second, a recent study using a mouse model of Duchenne muscular dystrophy demonstrated that AAV-mediated genome editing therapy resulted in undesirable integration of AAV genomes at a higher rate than previously considered^38^. These data raise caution regarding the premature therapeutic application of genome editing, and indicate the need for careful consideration and further studies. Nevertheless, the data presented in the current study provide a foundation for the future development of genome engineering in non-dividing cardiomyocytes.

## Materials and methods

### Cell culture and animal procedures

HEK293T cells were maintained in high glucose Dulbecco’s Modified Eagle Medium (DMEM, Gibco) containing 10% fetal bovine serum (FBS, Gibco) and penicillin, streptomycin and glutamine (PSG, Gibco). Neonatal mouse cardiomyocytes were prepared as previously described with modification^39^. In brief, harvested hearts were incubated in 0.025% trypsin/EDTA (Sigma) at 4 °C overnight and then digested with collagenase type II (Worthington). The cardiomyocyte fraction was collected after differential plating for 70 min at 37 °C, seeded and incubated with high glucose DMEM with 10% fetal bovine serum and PSG. The adhering cardiac fibroblast were separately incubated and used for experimental analysis after more than one passage. For animal procedures, a combination anesthetic was prepared with 0.3 mg/kg of medetomidine, 4.0 mg/kg of midazolam, and 5.0 mg/kg of butorphanol. The anesthetics were administered to 8-week old male Cas9 knock-in mice by intraperitoneal injection, and left lateral thoracotomy was performed under mechanical ventilation. For direct injection, 10 μl of AAV solution was directly injected into the left ventricular myocardium. For orbital injections, 100 μl of AAV solution was injected into the orbital venous sinus of the left or right eye. All procedures were performed in conformity with the *Guide for the Care and Use of Laboratory Animals published by the US National Institutes of Health* (NIH Publication, 8th Edition, 2011) and were approved by the Osaka University Committee for Laboratory Animal Use.

### gRNA and repair template constructs

gRNA and repair template constructs constructs were generated as previously described^9^. DNA sequences for 5′-terminal homology arm (5′-HA) and 3′-terminal homology arm (3′-HA) were amplified from genomic DNA using the primer pairs including recognition sites of restriction enzymes. DNA sequences for tdTomato fluorescent proteins was amplified from pCSCMV:tdTomato vector (Addgene #30,530). Each DNA fragment was digested by restriction enzymes and cloned into pUC19 vector (TaKaRa) to generate repair template. hU6 promoter and downstream guide RNA (gRNA) sequence was amplified from pX459 vector (Addgene #48,139). hU6 promoter sequence, gRNA sequence and repair template sequence were cloned into between inverse terminal repeat (ITR) sequences in pAAV vector (TaKaRa). PAM sequence mutation was introduced into each repair template to avoid Cas9-mediated cleavage. gRNA and primer sequences are listed in Supplementary Table.

### High-content image analysis

High-content image analysis was performed as previously described^9,40^. Briefly, cardiomyocytes (3 × 10^4^ cells/well) or cardiac fibroblasts (5 × 10^3^ cells/well) isolated from neonatal hearts of Cas9 knock-in mice were seeded in Greiner CELLSTAR 96-well plates precoated with collagen (Celmatrix Type I-C (KURABO)) one day before transduction with AAV or AdV encoding gRNA and repair template. Twenty-four h after primary culture, AAV or AdV encoding gRNA and repair template were transduced. Twelve h after transduction, medium were exchanged and cells were incubated for the indicated time. For sequential observation, fluorescent images from living cardiomyocytes cultured in 96-well plates were obtained by IN Cell Analyzer 6,000 (GE). After sequential observation, cells were fixed, immunostained and fluorescent images were obtained with the same imaging acquisition protocol. A total of 64 nonoverlap images (16 images per well) were obtained from each sample in one experiment using a 20x/0.45NA Nikon lens. The proportion of targeted integration was calculated as the ratio of tdTomato-positive cells per total cardiomyocytes using IN Cell Developer Toolbox (GE). The number of AdV-transduced cardiomyocytes in which tdTomato signals co-localized with sarcomeres was manually counted in all the acquired images because of these cells’ relatively low frequency.

### EdU labeling and imaging analysis

EdU labeling and imaging analysis were performed as previously described^9^. Briefly, murine neonatal cardiomyocytes (3 × 10^4^ cells/well) were seeded in Greiner CELLSTAR 96-well plates which were previously coated with collagen (Celmatrix Type I-C (KURABO)). Human iPSC-CMs (2 × 10^4^ cells/well) were replated in 96-well plates previously coated with gelatin (Nitta Gelatin). EdU was added to the culture medium from a 100 mM stock in DMSO (final concentration: 10 μM). After EdU labeling for the indicated time, cells were washed with PBS and fixed. The labeled EdU of the cells were visualized by Click-iT imaging kits (Life Technology) according to the manufacturer’s instructions. The samples were incubated with the working solution of Click-iT reaction cocktail, containing the Alexa Fluor azide and CuSO_4_, for 30 min at room temperature. After removal of the reaction cocktail, cells were washed with Click-iT reaction rinse buffer and PBS, then transferred to immunostaining procedures. After incubation with blocking buffer (1% BSA in PBS), the cells were immunostained with the indicated antibodies. Nuclei were stained with Hoechst. The immunofluorescent images were obtained using IN Cell Analyzer 6,000. A total of 16 nonoverlap images per well were obtained using a 20x/0.45NA Nikon lens. The obtained images were analyzed using IN Cell Developer toolbox (GE) as previously described^9^. For in vivo labeling, mice were treated with EdU at a dosage of 5 mg/kg body weight through intraperitoneal injections 2 h before AAV injections and for 5 consecutive days. Six days after AAV injections mice were sacrificed and heart samples were collected. EdU detection was carried out before immunofluorescent staining using the Click-iT imaging kits (Life Technology).

### Laser microdissection of heart tissue

Frozen sections of 10-μm thickness from heart cryoembedments were mounted onto RNase-free Polyethylene Naphthalate membrane frame slides (Leica 11,505,189). The sections were quickly fixed with cold acetone for 3 min, washed briefly in DEPC water, and stained with 0.05% toluidine blue solution for 1 min. Stained sections were then briefly rinsed 2 times in DEPC water and air dried for 10 min. tdTomato-positive cardiomyocytes were microscopically identified and isolated by laser microdissection at 20 × magnification using a LMD7000 Leica laser microdissection system. tdTomato-positive and -negative cardiomyocytes were pooled in 50 μl of QIAGEN RNeasy Micro Kit buffer dispensed centrally in the lid of a 0.5-ml Eppendorf cap. RNA was extracted according to the QIAGEN RNeasy protocol. Genomic DNA was extracted using a QIAAmp DNA micro kit (QIAGEN).

### Generation and purification of AAV

Generation and purification of AAV were performed as previously described^9^. To generate AAV2 or AAV6, HEK293T cells were transfected with the vector of interest, pHelper vector and pRC2-mi342 or pRC6 Vector (AAVpro Helper Free System, TaKaRa) using calcium phosphate transfection (CalPhos Mammalian Transfection Kit,TaKaRa). To generate AAV9, pAAV2/9n plasmid (Addgene #112,865, a gift from James M. Wilson) was used for packaging. Seventy-two h after transfection, HEK293T cells were detached by addition of 1/80 volume of 0.5 M EDTA (pH 8.0), then pelleted via low-speed centrifugation (2000 × g for 10 min). Cell pellet was lysed with AAV Extraction Solution A and centrifuged (9,000 × g for 10 min). AAV Extraction Solution B was added to the collected to supernatant and stored at -80 °C. Collected AAV generated from HEK293T cells (8–9 × 10^7^ cells) was purified using AAVpro Purification Kit (TaKaRa), and viral titer was calculated using AAV Titration Kit (TaKaRa) as previously described^9^.

### Generation and purification of AdV

Adenoviruses were generated using the ViraPower Adenovirus Expression System (Invitrogen). The transgene between ITRs of the AAV vector was subcloned into the pENTR/D-TOPO vector using a pENTR Directional TOPO Cloning Kit (Invitrogen). The DNA inserts were transferred to the pAd/CMV/V5-DEST vector via LR reaction with a Gateway System (Invitrogen). The plasmids were digested with Pac I (New England BioLabs), purified and transfected into subconfluent 293A cells using Lipofectamine 3,000 (Invitrogen). Then 293A cells were cultured for 10–12 days with replacement of the medium every 2 days. The cells and culture medium were harvested, freeze-thawed twice, and centrifuged to obtain the adenovirus-enriched supernatants. To amplify adenoviruses, the aliquots of the supernatants were added to cultured 293A. After 2 – 4 amplifications, the adenovirus-containing media were purified using an Adeno-X Maxi Purification Kit (TaKaRa). Viral titers were determined by a plaque-forming assay using 293A cells according to the manufacturer’s instruction.

### Human iPS cell generation and differentiation

The use of patient-derived samples was approved by the Ethics Committee of Osaka University hospital, and written informed consent was obtained and all research was performed in accordance with *Ethical Guidelines for Medical and Health Research Involving Human Subjects*. hiPSCs were generated from peripheral blood mononuclear cells (PBMCs) from a patient with cardiomyopathy. PBMCs were separated from peripheral whole blood using Ficoll-Plaque (GE). Reprogramming was performed by Sendai virus vectors with OCT3/4, SOX2, KLF4, and c-MYC (CytoTune-iPS 2.0 Sendai Reprogramming Kit, Life Technologies). hiPSCs were maintained in medium (StemFit AK02, AJINOMOTO) on a laminin-coated plate. hiPSCs were differentiated into cardiomyocytes according to a chemically defined protocol and maintained in medium supplemented with human albumin and ascorbic acid^25^.

## Supplementary information

Supplementary file1

Supplementary file2
