# Supplementary material for: Adeno-associated virus-mediated gene delivery promotes S-phase entry-independent precise targeted integration in cardiomyocytes
Source: Sci Rep. 2020 Sep 18;10:15348. doi: 10.1038/s41598-020-72216-y (PMC7501291; doi:10.1038/s41598-020-72216-y)

Supplementary Information

Adeno-associated Virus-mediated Gene Delivery Promotes S-phase Entry-independent Precise Targeted Integration in Cardiomyocytes

Authors/Affiliations

Yasuaki Kohama[1], Shuichiro Higo[1,2*], Yuki Masumura[1], Mikio Shiba[1], Takumi Kondo[1], Takamaru Ishizu[3], Tomoaki Higo[1], Satoki Nakamura[2,4], Satoshi Kameda[1], Tomoka Tabata[1], Hiroyuki Inoue[1], Daisuke Motooka[5], Daisuke Okuzaki[5], Seiji Takashima[4], Shigeru Miyagawa[6], Yoshiki Sawa[6], Shungo Hikoso[1] and Yasushi Sakata[1]

[1] Department of Cardiovascular Medicine, Osaka University Graduate School of Medicine, Suita, Osaka, 565-0871, Japan

[2] Department of Medical Therapeutics for Heart Failure, Osaka University Graduate School of Medicine, Suita, Osaka, 565-0871, Japan

[3] Higashiosaka City Medical Center, Higashiosaka, Osaka, 578-8588, Japan

[4] Department of Medical Biochemistry, Osaka University Graduate School of Medicine, Suita, Osaka 565-0871, Japan

[5] Genome Information Research Center, Research Institute for Microbial Diseases, Osaka University, Suita 565-0871, Japan

[6] Department of Cardiovascular Surgery, Osaka University Graduate School of Medicine, Suita, Osaka 565-0871, Japan

Corresponding Author

Shuichiro Higo, Associate Professor, Medical Therapeutics for Heart Failure

Department of Cardiovascular Medicine, Osaka University Graduate School of Medicine

Address: 2-2 Yamadaoka, Suita, Osaka 565-0871, Japan

Tel: +81-6-6879-3298, Fax: +81-6-6879-3299,

E-mail: higo-s@cardiology.med.osaka-u.ac.jp

Supplementary Materials and Methods

Reagents and antibodies

Antibodies used in this study: Cardiac Troponin I (Abcam Cat# ab47003 RRID:AB_869982), α-SMA (Sigma-Aldrich Cat# A2547 RRID:AB_476701), Troponin T-C (CT3) (Santa Cruz Biotechnology Cat# sc-20025, RRID:AB_628403). Alexa 488-, 568- and 647-conjugated secondary antibodies (Invitrogen).

Immunofluorescence

Cardiomyocytes were fixed with 4% PFA for 15 min at room temperature. After fixation, cells were permeabilized with 0.1% Triton-X 100 for 15 min, then incubated for 1 h with 1% BSA in PBS at room temperature. After blocking, cells were immunostained with primary antibodies diluted with 1% BSA. For secondary reaction, an Alexa 488-, 568- or 647- labeled secondary antibody (Invitrogen) was used. For tissue immunofluorescent staining, hearts were excised and immediately embedded in Tissue-Tek OCT cryo-embedding compound (Miles Laboratories). Cryostat sections at 5 μm were fixed in acetone for 10 min, rinsed 3 times in PBS and incubated with primary antibodies at 4 °C overnight after blocking with 1% BSA in PBS for 30 min. After washing with PBS, samples were stained with appropriate secondary antibodies for 1 h, and Nuclei were stained with Hoechst. Stained slides were rinsed with PBS and mounted in a FluorSave Reagent (Merck Millipore). Images were obtained by fluorescence microscope BZ-X700 (Keyence) and confocal laser scanning microscopy LSM710 (Carl Zeiss) or SpinSR10 (Olympus). Fluorescent images obtained by BX-X700 were processed and analyzed by BZ-X analyzer (Keyence).

RNA extraction and quantitative real-time PCR

Total RNA was extracted using RNeasy mini kit (QIAGEN) and converted to cDNA using High-Capacity cDNA Reverse Transcription Kit (Thermo Fisher Scientific). Quantitative real-time PCR was performed with THUNDERBIRD SYBR qPCR Mix (TOYOBO) using StepOnePlus Real-Time PCR Systems (Applied Biosystems). All of the samples were processed in duplicate. The level of each transcript was quantified by the threshold cycle method using Gapdh as an internal control.

Targeted deep sequencing

Genomic DNA were extracted both from neonatal cultured cardiomyocytes and adult heart tissues of Cas9 knockin mice treated with AAV6 encoding gRNA and repair template. Both experiments were performed in duplicate. The targeted DNA sequences were amplified with the primes outside the both 5'- and 3'- homology arms. NGS libraries were prepared from the amplified PCR products by the Nextera DNA Flex Library preparation kit (Illumina) and then sequenced using the Illumina Miseq with 251 bp paired end reads. After adapter trimming, sequence reads were mapped to the targeted genome of mouse *Myl2* with or without knocked-in tdTomato sequence by BWA 0.7.17[1]. The BAM format files were processed using igvtools[2] and the ratio of insertion, deletion or the ratio of knock-in of tdToamto were calculated as the average coverage at the corresponding sequence normalized by the average coverage at the total reference area.

Production of the lentivirus

shRNAs from the TRC1 library (Sigma-Aldrich, TRCN0000087763 and TRCN0000087765, referred to in the manuscript as shRNA#1 and shRNA#2, respectively) targeting Fanca and non-targeting shRNA control (SHC002, NTC) were used as transfer vectors. The lentivirus was produced by co-transfecting the transfer vector, third-generation packaging vector (pRSV-Rev, Addgene #12253; pMDLg/pRPE, Addgene #12251), and an envelope plasmid (pMD2.G, Addgene #12259) into subconfluent 293T cells using Lipofectamin 3000 (Invitrogen). Viral supernatants were collected 48 h after transfection and concentrated by centrifugation using PEG-it virus precipitation solution (System Biosciences). Aliquots were reconstituted in phosphate-buffered saline (PBS) and stored at −80 °C until use. Lentiviral particles were used to transduce cardiomyocytes in the presence of 8 μg/ml polybrene.

Generation of Cas9 mRNA and transfection

The Cas9 mRNA was transcribed using FLAG-tagged Cas9 expression vector and the mMESSAGE mMACHINE T7 ULTRA kit (Life Technologies). After in vitro transcription, DNase I treatment and poly (A) tailing reaction were performed. The generated RNAs were then purified using MEGA clear kit (Life Technologies). Purified mRNA was

transfected into hiPSCs using Lipofectamine messenger MAX (Life Technologies).

Statistical Analysis

Data are expressed as means ± S.D. of at least three independent experiments, unless otherwise indicated. Statistical analysis was performed using the JMP (SAS, NC). The two- tailed Student's t-test was used to analyze differences between two groups. p-value less than 0.05 was considered statistically significant.

1 Li, H. & Durbin, R. Fast and accurate short read alignment with Burrows-Wheeler transform. *Bioinformatics* **25**, 1754-1760, doi:10.1093/bioinformatics/btp324 (2009).

2 Robinson, J. T. *et al.* Integrative genomics viewer. *Nat Biotechnol* **29**, 24-26, doi:10.1038/nbt.1754 (2011).

Supplementary Figure Legends

Supplementary Figure 1

Evaluation of AAV-mediated targeted integration in *in vitro* cardiomyocytes and *in vivo* heart tissues.

A) Neonatal cardiomyocytes isolated from Cas9 knock-in mice were seeded in 96-well plates and transduced with AAV9 (3.1 x 10^5 viral genomes/cell). Five days after transduction, cells were fixed and stained with anti-troponin I antibody (green). Nuclei were stained with Hoechst. Cardiomyocytes in white squares are enlarged in the right panels. Scale bar: 100 μm.

B) AAV6 (2.5 x 10^{10} viral genome/100 μl) or AAV9 (9.2 x 10^{11} viral genome/100 μl) was systemically injected via the orbital venous sinus into 8-week-old Cas9 knock-in mice. Seven days after injection, heart tissues were excised and PCR was performed using genomic DNA samples obtained from the heart tissues. PCR primers were designed as shown in Fig. 1B. Genomic DNA obtained from heart tissue sections without viral injections were used as control. Original full image of the gel is presented in Supplementary Figure 6F.

C) Seven days after systemic injection of AAV6 or AAV9, heart tissues were excised. The frozen sections of heart tissue were fixed and stained with anti-troponin I antibody (green) and Hoechst. Scale bar: 300 μm.

D) The PCR band shown in Fig. 1D was excised, purified, and analyzed by direct Sanger sequencing.

E) AAV6 (2.5 x 10^9 viral genome/10 μl) was directly injected into the free left ventricular wall of 8-week-old WT mice. Five days after injection, heart tissues were excised and stained with anti-troponin I antibody (green). Scale bar: 300 μm.

F) 5-ethynyl-2'-deoxyuridine (EdU) was intraperitoneally injected into Cas9 knock-in mice 2 h before the direct injection of AAV6 into the myocardium, then administrated for 5 consecutive days after injection.

G) At day 5 the frozen sections of heart tissue were fixed and immunostained with anti-troponin I antibody (white). The incorporated EdU (green) in the cells was visualized using Click-iT imaging kits. tdTomato-positive cells in the white rectangle are enlarged in the right panels. Scale bar: 20 μm.

Supplementary Figure 2

Evaluation of AAV-mediated targeted integration in *in vivo* heart tissues.

A) The PCR band shown in Fig. 2C was excised, purified, and analyzed by direct Sanger sequencing.

B) Tissue sections obtained at day 28 after AAV6 injection were immunostained with anti-troponin I antibody (green) and high-magnification image was obtained by confocal microscopy. Scale bar: 10 μm.

C) Cardiac sections that included tdTomato-positive cardiomyocytes at the injected site and tdTomato-negative cardiomyocytes at the remote site were dissected by laser microdissection. Scale bar: 1 mm.

D) Genomic DNA was extracted from tdTomato-negative cardiomyocytes at a remote site. PCR was performed using the primers to amplify the transgenes (Fig. 1B). Original full image of the gel is presented in Supplementary Figure 6G.

Supplementary Figure 3

Evaluation of AdV-mediated targeted integration in cardiac fibroblasts and cardiomyocytes.

A) The mouse *Actb* gene consists of 6 exons (upper). The repair template consists of the tdTomato sequence between the 680-bp 5'-terminal and the 776-bp 3'-terminal homology arms (5'-HA and 3'-HA, respectively), corresponding to the genomic sequence of the 3'-terminus of *Actb*. A PAM mutation (CC to AA) was introduced into 5'-HA, and the stop codon sequence was removed. The expected genomic sequence of the Actb-tdTomato fusion gene after successful targeted integration is shown in the lower panel. Arrows indicate the locations of the PCR primers (the forward primer was designed outside 5'-HA).

B) A DNA sequence containing hU6 promoter (hU6p)–driven gRNA and the tdTomato repair template in the opposite direction was subcloned into AdV.

C) Neonatal cardiac fibroblasts isolated from Cas9 knock-in mice were seeded in 96-well plates and transduced with AdV. Five days after transduction, cells were fixed and stained with anti-α-SMA antibody (green). Nuclei were stained with Hoechst. Scale bar: 50 μm.

D) Cardiac fibroblasts were treated as in (C). Five days after transduction, genomic DNA was extracted and the

targeted sequence was amplified by PCR using the primers indicated in (A). Original full image of the gel is presented in Supplementary Figure 6H.

E) The PCR band shown in (D) was excised, purified, and analyzed by direct Sanger sequencing.

F) AdV (1.4 x 10^{10} PFU/ml, 10 μl) encoding gRNA and repair template was directly injected into the free left ventricular wall of 8-week-old Cas9 knock-in mice. Five days after injection, heart tissues were excised. The frozen sections of heart tissue were fixed and stained with anti-troponin I antibody (green) and Hoechst. Scale bar: 50 μm.

G) PCR was performed using genomic DNA samples obtained from the heart tissues injected with AAV or AdV encoding gRNA and repair template. PCR primers were designed as shown in Fig. 1B. Original full image of the gel is presented in Supplementary Figure 6I.

H) Neonatal cardiomyocytes isolated from Cas9 knock-in mice were transduced with AAV6 (8.3 x 10^4 viral genomes/cell) or AdV (1.4 x 10^{10} PFU/ml, 1 μl/well) encoding gRNA targeting the 3' end of the mouse *Myl2* gene and repair template DNA. Five days after transduction, quantitative real-time PCR was performed using the extracted DNA as PCR templates. Forward and reverse primers were designed to amplify the internal sequence of tdTomato shown in Fig. 1B. As internal control, Ct (cycle threshold) values were calculated from the primer pairs used to amplify the genomic locus of *Gapdh*. The relative values normalized by the AAV6 copy number are shown (n=3, means ± SD).

I) The PCR bands shown in Fig. 3D were excised, purified, and analyzed by Sanger sequencing.

Supplementary Figure 4

shRNA-mediated knockdown and gRNA-mediated NHEJ targeting *Fanca*.

A) Neonatal cardiomyocytes isolated from Cas9 knock-in mice were seeded in 24-well plates and transduced with lentivirus encoding shRNA against *Fanca*. Non-target shRNA was used as control. Two days after transduction, total RNA was extracted and converted to cDNA. The mRNA expression levels of *Fanca* in cardiomyocytes were analyzed by quantitative real-time PCR (n=4, means ± SD). *: $p<0.01$ vs control.

B) Neonatal cardiomyocytes isolated from Cas9 knock-in mice were seeded in 24-well plates and transduced with AAV6 encoding gRNA targeting *Fanca*. Two days after transduction, genomic DNA was extracted and the targeted sequence was amplified by PCR. The PCR band was excised, purified, and analyzed by Sanger sequencing. Representative sequencing results are shown.

C) Neonatal cardiomyocytes isolated from Cas9 knock-in mice were seeded in 24-well plates and transduced with AAV6 encoding gRNA targeting *Fanca*. gRNA targeting LacZ was used as control. Two days after transduction, total RNA were extracted and converted to cDNA. The mRNA expression levels of *Fanca* in cardiomyocytes were analyzed by quantitative real-time PCR (n=4, means ± SD). *: $p<0.05$, vs control.

D) Neonatal cardiomyocytes isolated from Cas9 knock-in mice were seeded in 24-well plates and transduced with lentivirus encoding shRNA against *Brca2*. Non-target shRNA was used as control. Two days after transduction, total RNA was extracted and converted to cDNA. The mRNA expression levels of *Brca2* in cardiomyocytes were analyzed by quantitative real-time PCR (n=5, means ± SD). *: $p<0.01$ vs control.

E) Cardiomyocytes were treated as in Fig. 4A using shRNA against *Brca2*. Cells were stained with anti-troponin I antibody (green). Scale bar: 100 μm. The proportion of tdTomato-positive cardiomyocytes was calculated using high-content image analysis (n=7, means ± SD). *: $p<0.01$ vs control.

Supplementary Figure 5

Evaluation of AAV-mediated targeted integration in human iPSC-derived cardiomyocytes.

A) Ten days after cardiomyocyte differentiation of hiPSCs, the proportion of TNNT2-positive cells were calculated by FACS analysis (n=3, means ± SD).

B) Ten days after cardiomyocyte differentiation, hiPSC-CMs were trypsinized and replated into 96-well plates pre-coated with gelatin. AAV2 encoding EGFP (1.04×10^4 viral genome/cell) was transduced into hiPSC-CMs at day 14 after differentiation. Five days after transduction, hiPSC-CMs were fixed and immunostained with anti-troponin T antibody (white). NTD indicates non-transduction control. Scale bar: 50 μm.

C) Differentiated hiPSC-CMs seeded in 96-well plates were incubated with 5 μM EdU for 72 h, then cells were

fixed and immunostained with anti-troponin T antibody (white). Scale bar: 100 μm.

D) The proportions of EdU-positive hiPSC-CMs were determined by high-content image analysis (n=3, means ± SD).

E) The PCR band shown in Fig. 5D was excised, purified, and analyzed by Sanger sequencing.

Supplementary Figure 6

Original full images of gels

A) Corresponds to Fig. 1D

B) Corresponds to Fig. 2C

C) Corresponds to Fig. 3C

D) Corresponds to Fig. 3D

E) Corresponds to Fig. 5D

F) Corresponds to Supplementary Fig. 1B

G) Corresponds to Supplementary Fig. 2D

H) Corresponds to Supplementary Fig. 3D

I) Corresponds to Supplementary Fig. 3G

Boxes highlight the cropped bands reported in each figure.

Supplementary Table

List of oligonucleotides, gRNA and shRNA used in the study.

		**sequence (5'-3')**	**Application**
Mm_Actb_KI_tdT_F	primer	atcctgaccgagcgtggctaca	Confirmation of tdTomato knock-in at Actb locus

Mm_Actb_KI_tdT_R	primer	tgccatgccccaggaacaggtg	Confirmation of tdTomato knock-in at Actb locus
Mm_Myl2_KI_tdT_F	primer	tgagctcatggagatccacc	Confirmation of tdTomato knock-in at Myl2 locus
Mm_Myl2_KI_tdT_R	primer	gtagatggtcttgaactccacca	Confirmation of tdTomato knock-in at Myl2 locus
Mm_Myl2_CDS_F	primer	cacacaagcagagaggttctccaa	Confirmation of tdTomato knock-in at Myl2 locus (cDNA)
Mm_Myl2_CDS_KI_tdT_R	primer	tgccatgccccaggaacaggtg	Confirmation of tdTomato knock-in at Myl2 locus (cDNA)
tdT_F	primer	gccaccacctgttcctggggcat	amplify tdTomato transgene by qRT-PCR
tdT_R#1	primer	tgacggccatgttgttgtcctcgga	amplify tdTomato transgene by qRT-PCR
Mm_Myl2_F	primer	tgagctcatggagatccacc	amplify Myl2 target sequence for deep sequencing
Mm_Myl2_R	primer	cagcctcagtgtctctggaagagc	amplify Myl2 target sequence for deep sequencing
BamHI_Mm_Myl2_F	primer	GGATCCaacacgtggacccagaagtt	PCR confirmation of tdTomato transgene
tdT_R#2	primer	gtagatggtcttgaactccacca	PCR confirmation of tdTomato transgene
Fanca_shRNA#1	shRNA	ccggtgcacccatctcagcgcaccactcgagtggtgcgctgagatgggtgcatttttg	shRNA sequence targeting Fanca
Fanca_shRNA#2	shRNA	cgggccaccaaagttcgaggagttactcgagtaactcctcgaactttggtggtttttg	shRNA sequence targeting Fanca
Brca2_shRNA	shRNA	ccgggcagtcaatatgaactctgaactcgagttcagagttcatattgactgctttttg	shRNA sequence targeting Brca2
Mm_Fanca_sgRNA	sgRNA	gagataccatcaaaatatgc	sgRNA sequence targeting exon 2 of Fanca
Mm_Fanca_F1	primer	gcggtttcttactgtttgtgc	To analyze mRNA expression levels of

			Fanca by qRT-PCR
Mm_Fanca_R1	primer	acaatcttaacacaacgaactgaaa	To analyze mRNA expression levels of Fanca by qRT-PCR
Mm_Brca2_F1	primer	gactttgtttaattcgagaactaggaa	To analyze mRNA expression levels of Brca2 by qRT-PCR
Mm_Brca2_R1	primer	tcttttgattgggggttgtc	To analyze mRNA expression levels of Brca2 by qRT-PCR
Mm_Gapdh_F	primer	tcaacggcacagtcaagg	To analyze mRNA expression levels of Gapdh by qRT-PCR
Mm_Gapdh_R	primer	cacgacatactcagcacc	To analyze mRNA expression levels of Gapdh by qRT-PCR
Mm_Fanca_F2	primer	ctaggggtgcctgccatgggt	Confirmation of NHEJ at Fanca locus
Mm_Fanca_R2	primer	ggcaccagttactcgccaagt	Confirmation of NHEJ at Fanca locus
Hs_MYL2_stop	sgRNA	actctgcaaagacgagccca	sgRNA sequence targeting 3'-terminal of human MYL2
Hs_MYL2_KI_tdT_F	primer	tgccaaagcttctgtatgagcaagc	Confirmation of tdTomato knock-in at human MYL2 locus
Hs_MYL2_KI_tdT_R	primer	tgccatgccccaggaacaggtg	Confirmation of tdTomato knock-in at human MYL2 locus

Supplementary Movie

Fluorescent-labeled sarcomeres in monolayered hiPSC-CMs

Supplementary Figure 1

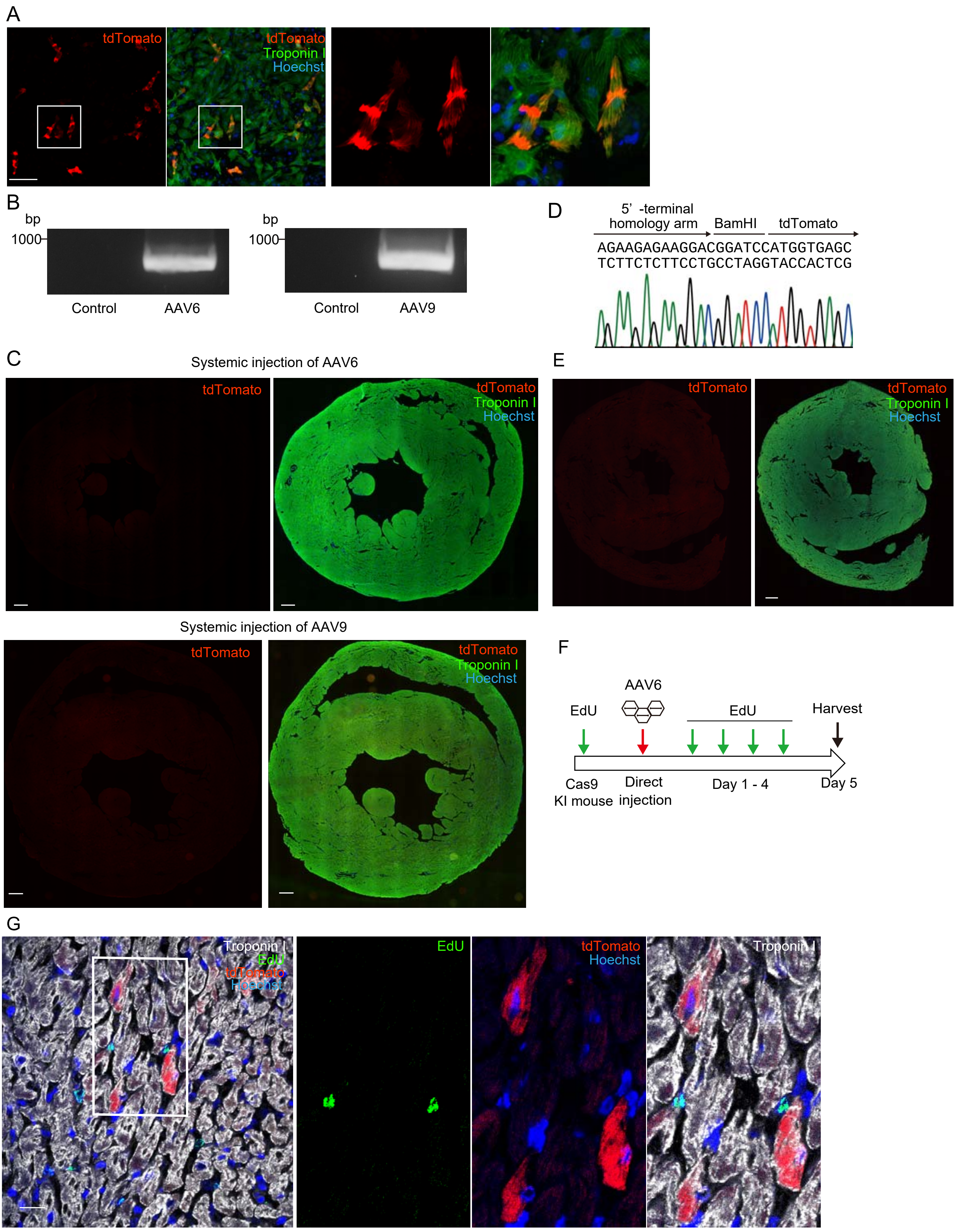

Supplementary Figure 2

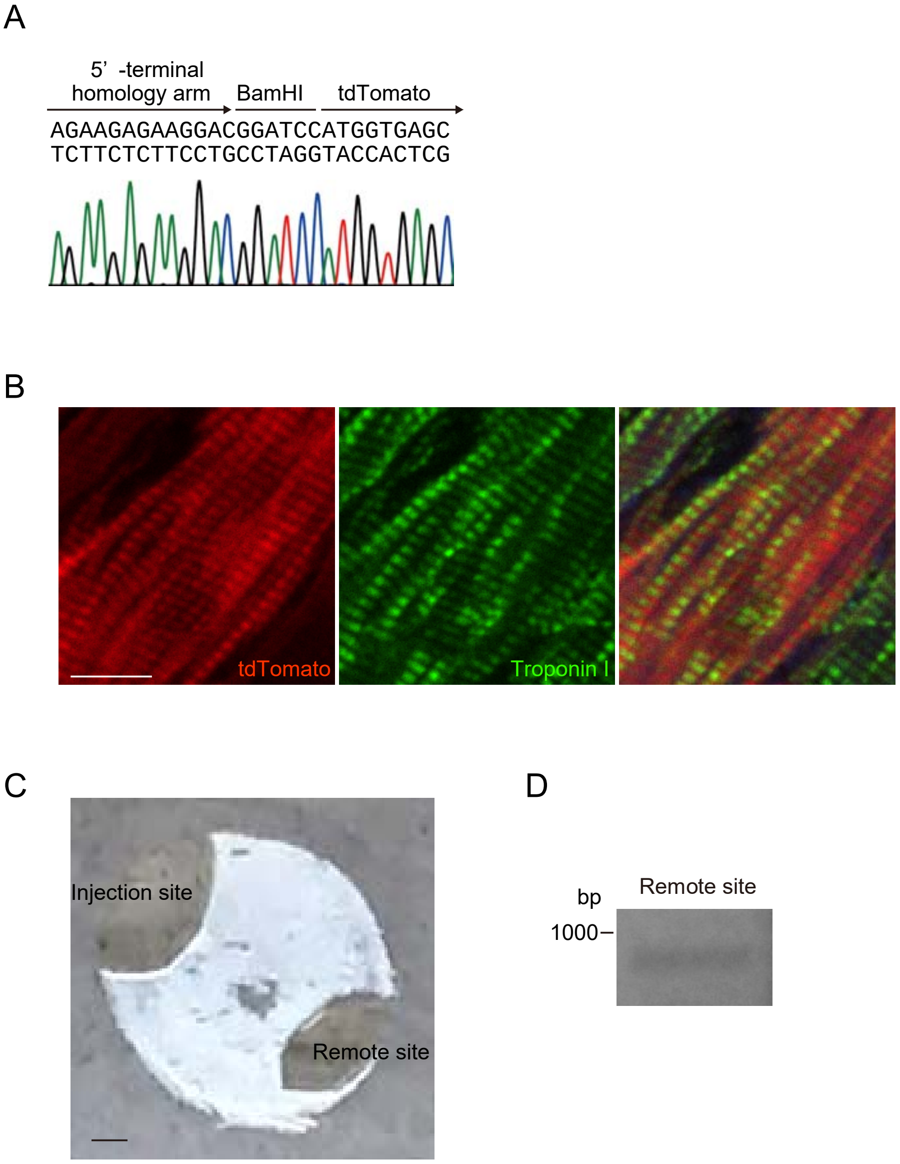

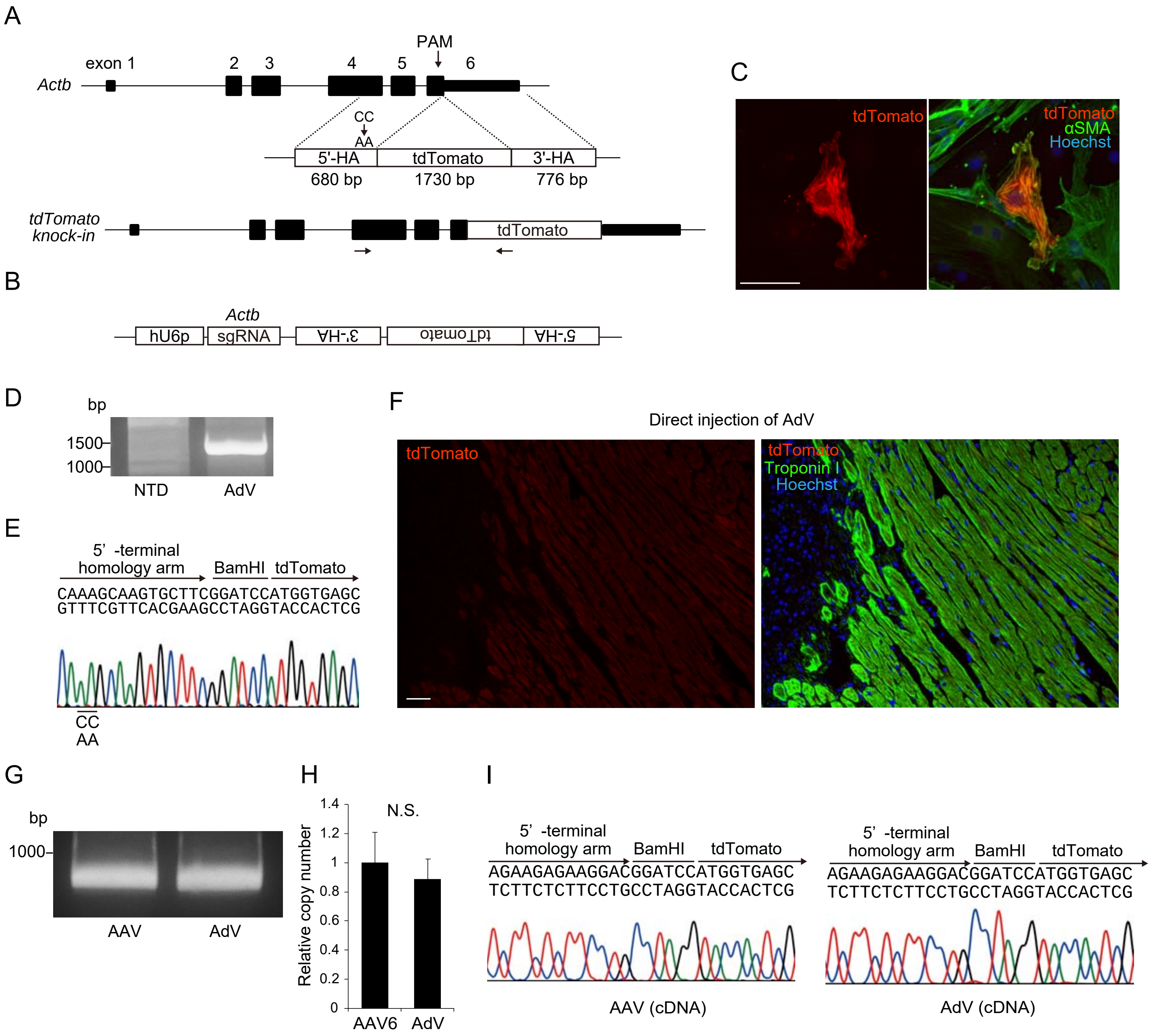
A
exon 1
2
3
4
5
PAM
6
Actb
CC
AA
5'-HA
tdTomato
3'-HA
680 bp
1730 bp
776 bp
tdTomato knock-in
tdTomato
B
Actb
hU6p
sgRNA
C
tdTomato
tdTomato
αSMA
Hoechst
D
bp
1500
1000
NTD
AdV
E
5' -terminal homology arm
BamHI
tdTomato
CAAAGCAAGTGCTTCGGATCCATGGTGAGC
GTTTCGTTCACGAAGCCTAGGTACCACTCG
CC
AA
F
Direct injection of AdV
tdTomato
tdTomato
Troponin I
Hoechst
G
bp
1000
AAV
AdV
H
N.S.
Relative copy number
1.4
1.2
1
0.8
0.6
0.4
0.2
0
AAV6
AdV
I
5' -terminal homology arm
BamHI
tdTomato
AGAAGAGAAGGACGGATCCATGGTGAGC
TCTTCTCTTCCTGCCTAGGTACCACTCG
AAV (cDNA)
AdV (cDNA)

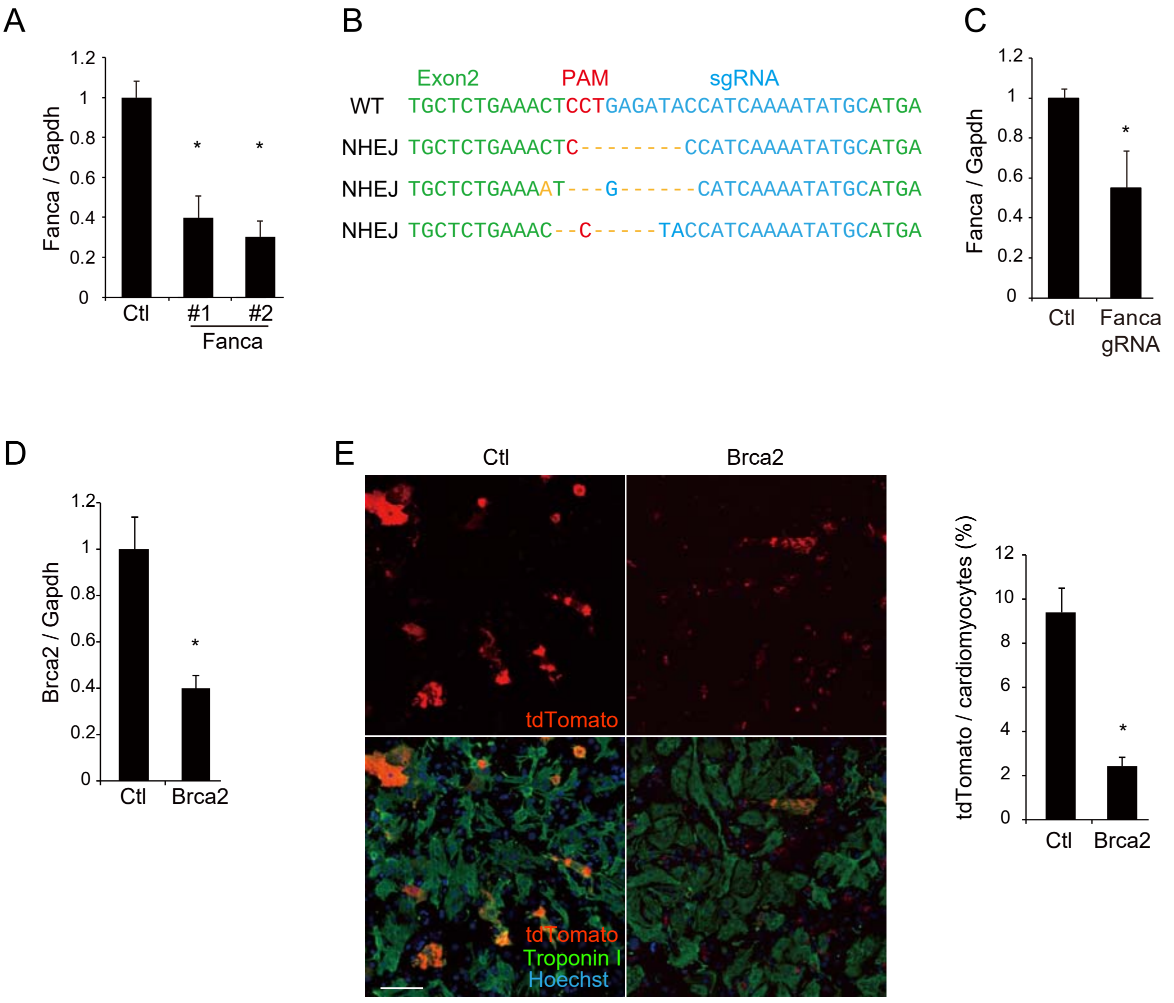
A
1.2
1
0.8
0.6
0.4
0.2
0
Fanca / Gapdh
*
*
Ctl
#1
#2
Fanca
B
Exon2
PAM
sgRNA
WT TGCTCTGAAACTCCTGAGATACCATCAAAATATGCATGA
NHEJ TGCTCTGAAACTC--------CCATCAAAATATGCATGA
NHEJ TGCTCTGAAAAT---G------CATCAAAATATGCATGA
NHEJ TGCTCTGAAAC--C-----TACCATCAAAATATGCATGA
C
Fanca / Gapdh
*
Ctl
Fanca gRNA
D
Brca2 / Gapdh
*
Ctl
Brca2
E
Ctl
Brca2
tdTomato
tdTomato
Troponin I
Hoechst
tdTomato / cardiomyocytes (%)
12
10
8
6
4
2
0
*
Ctl
Brca2

Supplementary Figure 5

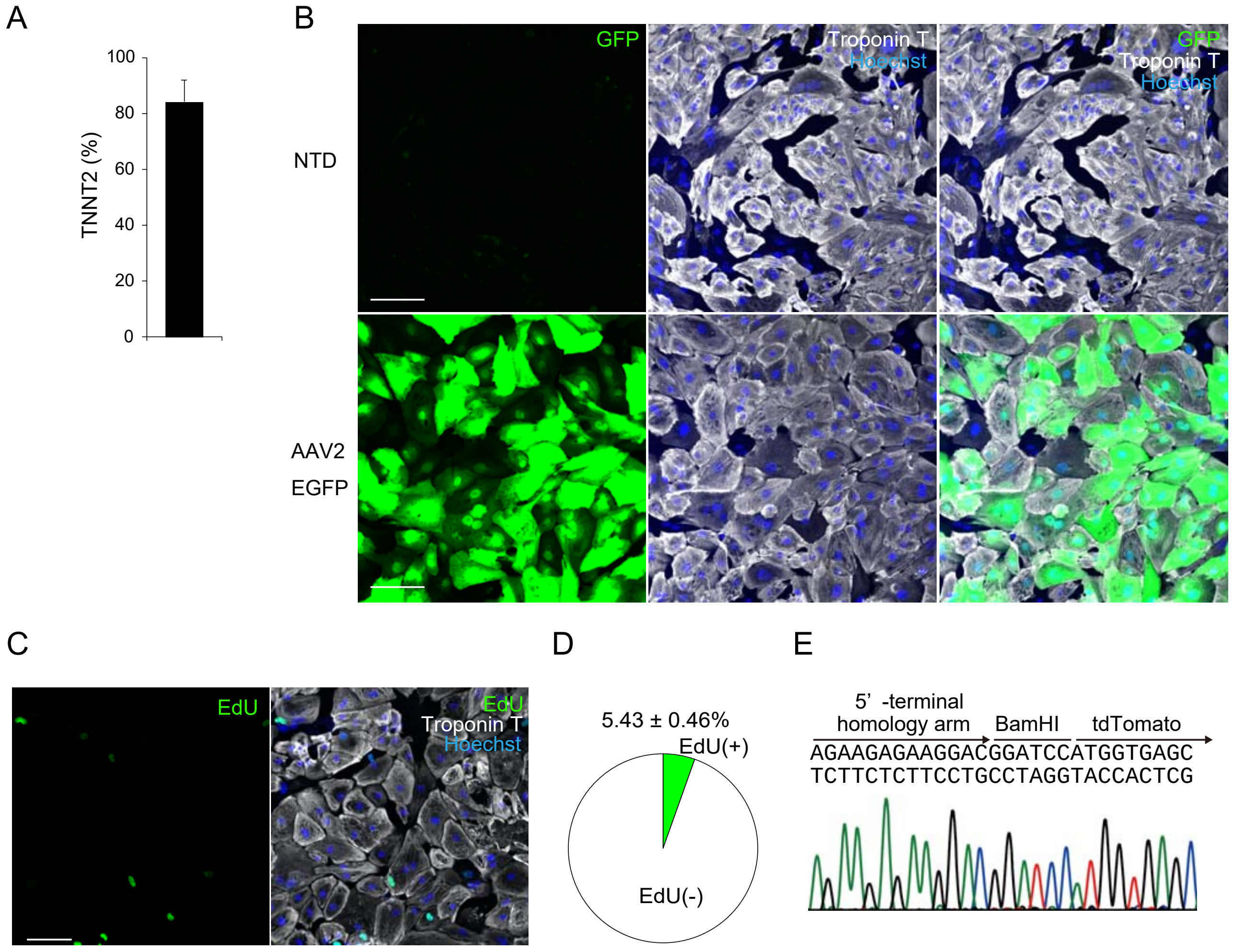

Supplementary Figure 6

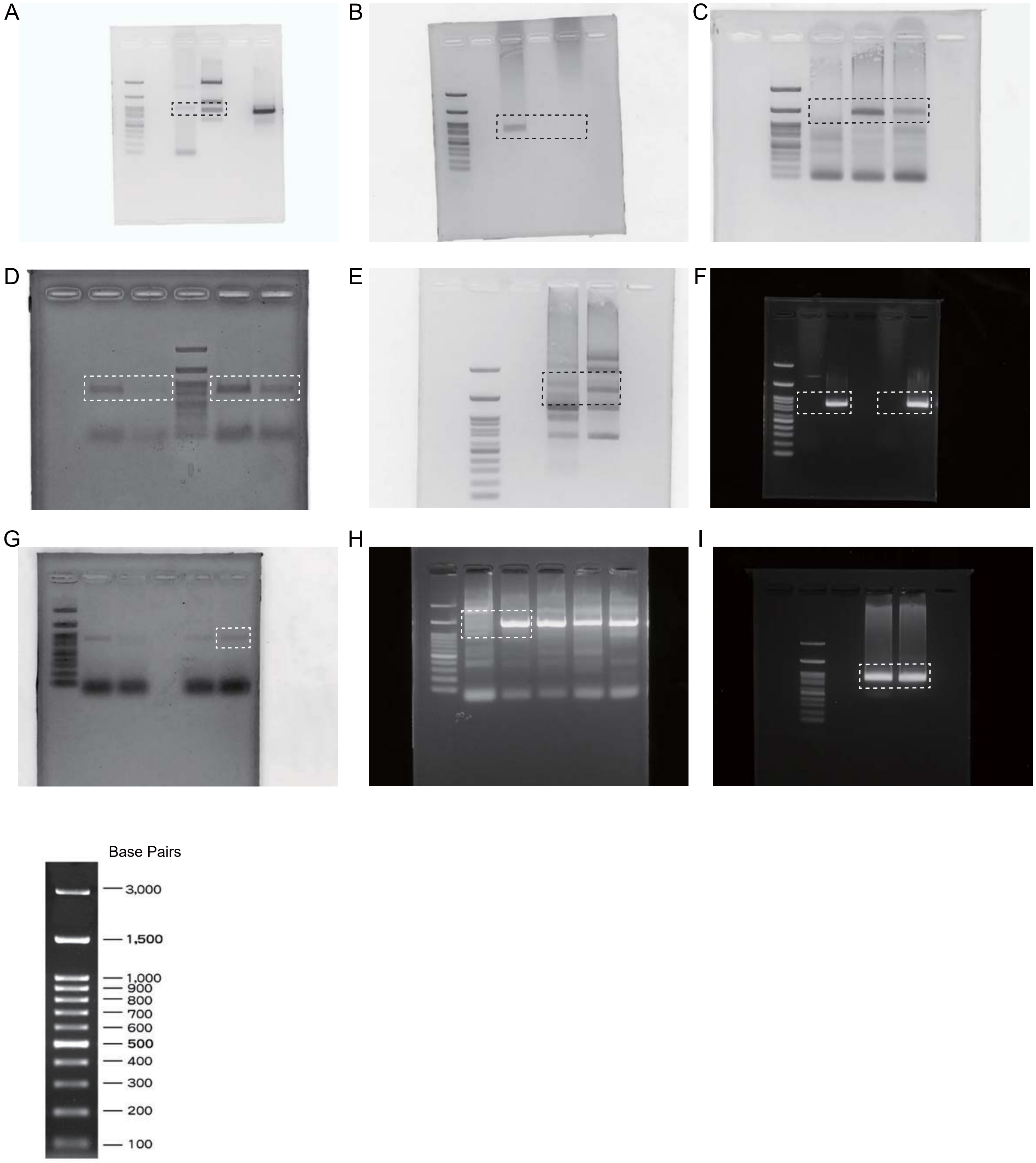

Supplement: Supplementary file 1 — Supplementary file1 [file 41598_2020_72216_MOESM1_ESM.pdf]
